# Supplementary material for: Neuronal tau pathology worsens late-phase white matter degeneration after traumatic brain injury in transgenic mice
Source: Acta Neuropathol. 2023 Aug 14;146(4):585–610. doi: 10.1007/s00401-023-02622-9 (PMC10499978; doi:10.1007/s00401-023-02622-9)
Supplement: Supplementary file 1 — Supplementary file1 (PDF 1577 KB) [file 401_2023_2622_MOESM1_ESM.pdf]

Supplemental Information (SI)

Supplemental Information Table SI-1: ***Selected relevant studies of TBI in human tau mouse lines*****.** Recent systematic reviews provide a more comprehensive discussion of tau pathology after TBI in wildtype and transgenic mice (Bachstetter et al., 2020; Kahriman et al., 2021).

**human tau mouse line** (source citation)	**Tau protein**	**promoter**	**genotype control mice**	**TBI model (age at time of TBI)**	**post-injury time points**	**sex**	**TBI study citation**
**hTau.P301S (Tg2541)** B6-Tg(Thy1-MAPT*P301S)2541 (Allen et al., 2002; Johnson et al., 2017)	human P301S mutation in 0N4R hTau isoform (hemizygous and homozygous)	murine Thy1.2 (neuronal)	wild type littermates	moderate **CHI** 1x (8 wks)	1 dpi, 6 wpi, and 4 mpi	♂ ♀	Current Study
			wild type littermates	mild **r-CHI** 1/dy x 5 dys (8 wks)	1 dpi, 6 wpi, and 4 mpi	♂ ♀	Current Study
**hTau58.4** (Yin et al., 2017)	human P301S mutation in 0N4R hTau isoform (homozygous)	murine Thy1.2 (neuronal)	C57BL/6	mild **r-CHI** 6/dy x 7 dys (12-16 wks)	1 and 3 mpi	♂	Cheng et al., 2020
**hTau.P301S (PS19)** B6;C3-Tg(Prnp-MAPT*P301S)PS19Vle/J (Yoshiyama et al., 2007)	human P301S mutation in 1N4R hTau isoform (homozygous)	murine prion protein	wild type littermates	moderate-severe **CCI** 1x (12 wks)	1 dpi, 1 wpi, 2 and 6 mpi	not stated	Edwards et al., 2020
			C57BL/6J	mild **r-CHI** 1/dy x 3 (35-41 dys)	40 dpi	♂	Izzy et al., 2021
**hTau** B6.CgMapt$^{tm1(EGFP)Klt}$ Tg(MAPT)8cPdav/J (Andorfer et al., 2003)	human MAPT 6 hTau isoforms (hemizygous) and murine MAPT knockout (homozygous)	human MAPT	none	mild **r-CHI** 1x and 5x over 10 dys (12 wks)	acute, 6 and 12 mpi	♂	Mouzon et al., 2019
				Mild **r-CHI** 1/dy x 20 (16 wks)	acute, 3 and 12 mpi	♂	Gangolli et al., 2019

MAPT = microtubule-associated protein tau
CHI = concussive closed head injury; r-CHI = repetitive CHI
CCI = controlled cortical impact onto dura (with craniotomy)
mpi, wpi, or dpi = months, weeks, or days post injury as reported by study

Supplemental Information Figure SI-1. ***Weights of mice included in hang time assessment of neurologic deficits.*** See legend to Figure 1.

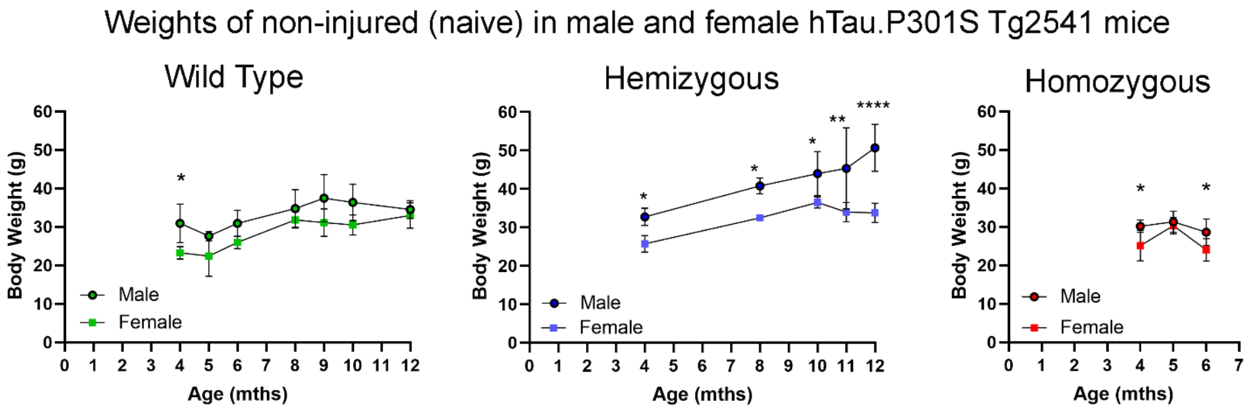

Supplemental Information Figure SI-2. ***Righting reflex recorded immediately after r-sham or r-mTBI procedures with data shown for all five days for mice of each genotype.*** See legend to Figure 1.

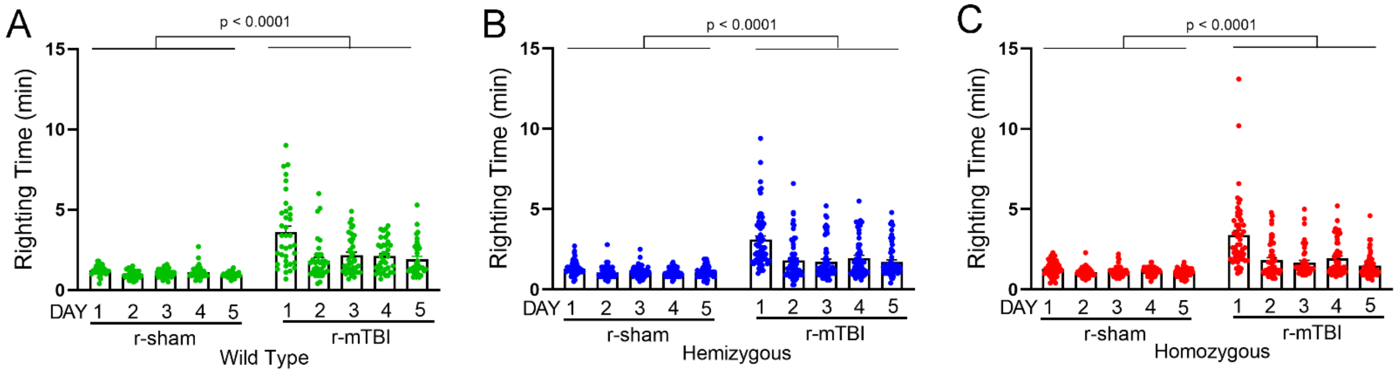

Supplemental Information Figure SI-3. ***Acute axon damage in the corpus callosum at 24 hours after the moderate single (s-TBI) is more extensive than after the repetitive mild TBI (r-mTBI).*** See Figure 1.

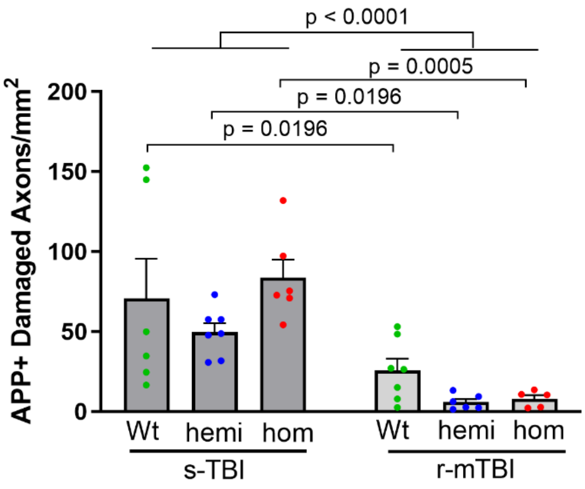

Supplemental Information Figure SI-4. *Assessment of social impairment using the 3-chamber assay*.

Methods for social interaction assessment

This r-mTBI model produced social interaction deficits in C57BL/6 adult mice in our prior study (Yu et al., 2017). Therefore, mice of the P301S line were tested for changes associated with TBI or genotype. The social interaction assessment was carried out using a three-chamber sociability test apparatus (Stoelting Co., Wood Dale, IL). The test mouse was first placed in the middle chamber and allowed 10 minutes to explore all chambers for habituation to the apparatus. A small wire enclosure was placed in each of the side chambers and the mouse was allowed to explore all chambers for 10 minutes to habituate to the enclosures as novel objects. Social approach was then tested by placing an unfamiliar C57BL/6J mouse (stranger) of the same sex and age into one of the wire enclosures. The test mouse was again placed into the middle chamber and allowed to explore among the three chambers for 10 minutes. The movement of the test mouse was video recorded and then manually scored to determine the time spent exhibiting social approach behavior (defined as movement toward, circling, or sniffing of the stranger mouse in the wire enclosure) using ANY-Maze software (Stoelting Co.).

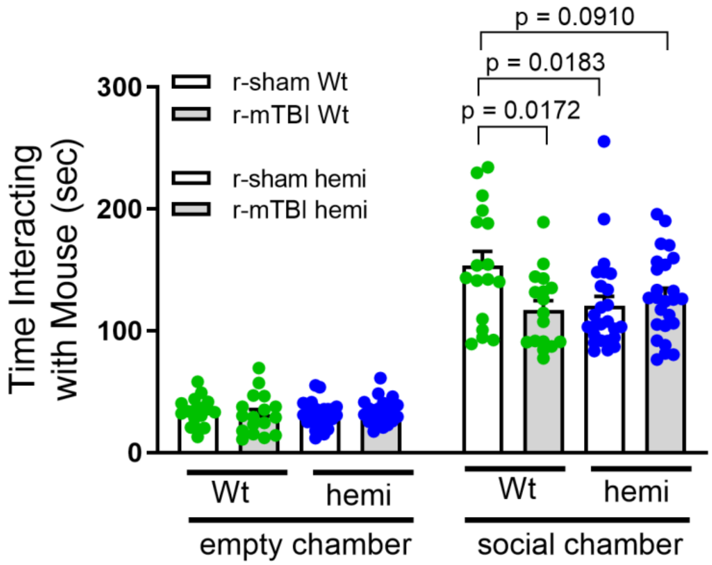

Figure SI-4. *Assessment of social impairment using the 3-chamber assay*. After habituation to a central zone, doors are open to two adjacent chambers for the test mouse to choose either the empty chamber with an empty wire carrier or the social chamber with a non-familiar mouse in the wire carrier. Mice of all conditions

exhibit a clear preference of entering the social chamber to interact with the non-familiar mouse. The wild type (Wt) injured mice (r-mTBI; Wt, gray bars) spend significantly less time interacting with the non-familiar mouse, as compared to the interaction time for the sham mice (r-sham; Wt, white bars). With hemizygous hTau.P301S Tg2541 mice (hemi), both the r-sham mice exhibit reduced time interacting with the non-familiar mouse, indicating that the tau expression produces social impairment. Male and female mice were tested as separate cohorts. Mice received the injury or sham procedures at 8 weeks of age and were for social testing was conducted 6 weeks later. One-way ANOVA with Dunnett’s test.

Supplemental Information Figure SI-5. ***Neuroinflammation in the medial cortex under the impact site*.**

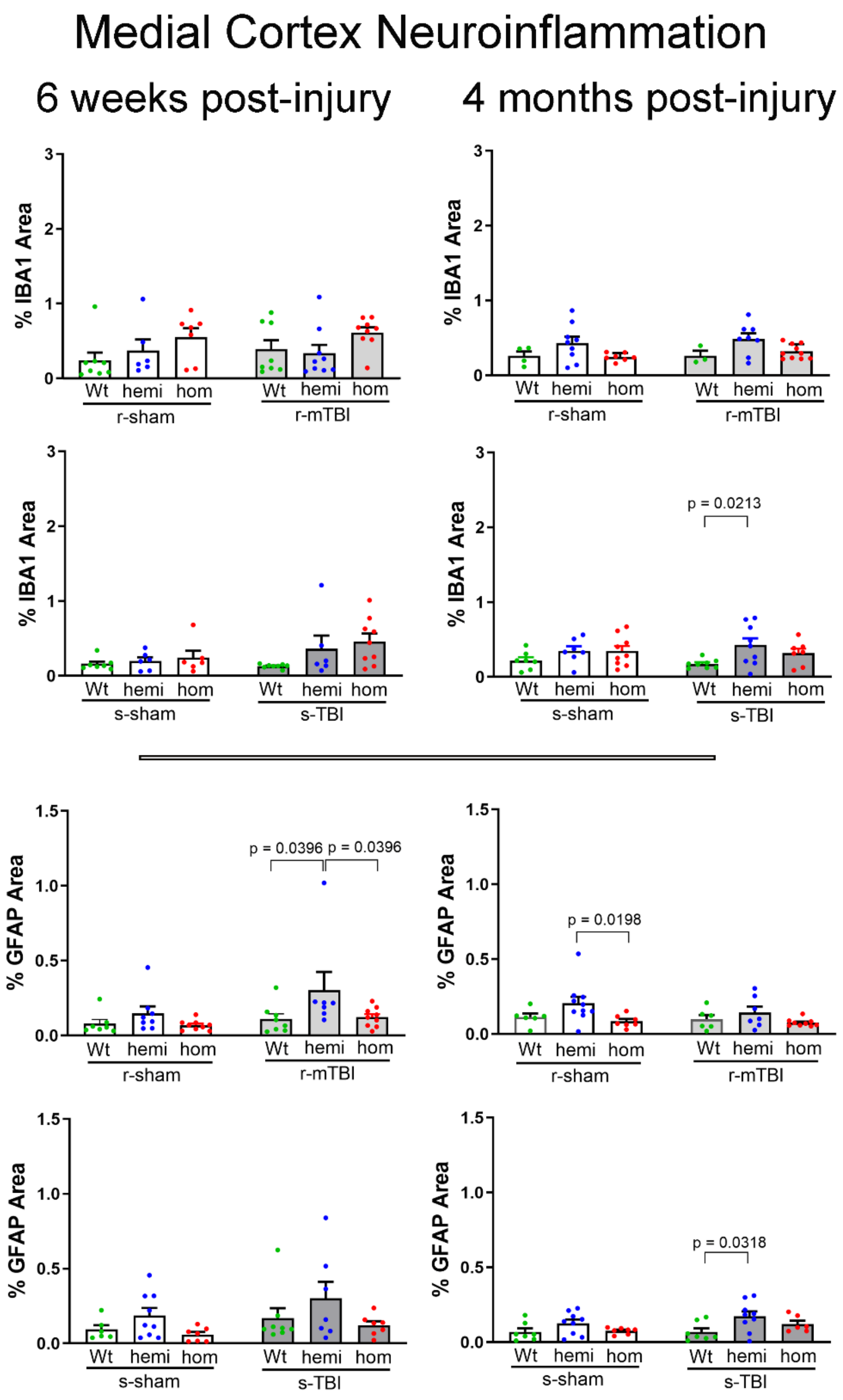

Supplemental Information Figure SI-6. ***AT8 immunolabeling in spinal cord sections.***

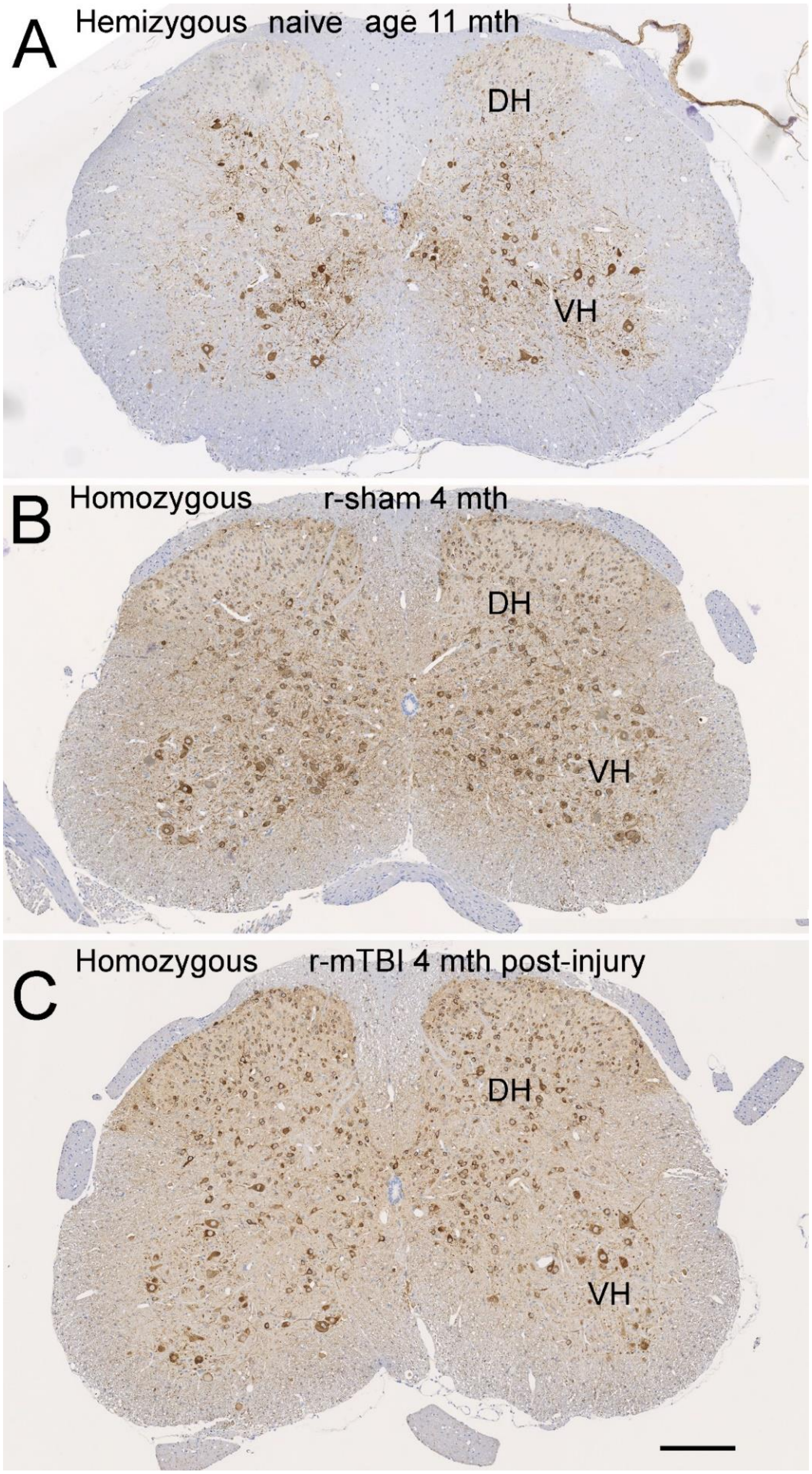

Figure SI-6. ***AT8 immunolabeling in spinal cord sections.*** Phosphorylated tau accumulations are seen in sensory neurons of the dorsal horn (DH) and motor neurons of the ventral horn (VH). These examples illustrate tau from regions outside the injury site contribute to total human tau in serum samples. Scale bars = 200 µm.

Supplemental Information Table SI-2 and S3. Weights and hang time testing of non-injured (naïve) mice. Statistical analysis for combined male and female mice across hTau.P301S genotypes for Tg2541 mice. Further analysis was conducted for sex as a biological variable for groups with 3 or more mice, with gray text for excluded comparisons that do not have at least 3 mice of both sexes.

**SI-2 Hang Time**	**Main effect; F, p-value**		**Adjusted p-value**	
Non-injured mice<br>WT, Hemi, Hom		Log-rank (Mantel-Cox) test	genotype effect	**p<0.0001**
WT<br>♂ (n = 11)<br>♀ (n = 8)		Log-rank (Mantel-Cox) test	WT male vs. female	p=0.9191
Hemi<br>♂ (n = 7)<br>♀ (n = 8)		Log-rank (Mantel-Cox) test	Hemi male vs. female	p=0.2246
Hom<br>♂ (n = 15)<br>♀ (n = 4)		Log-rank (Mantel-Cox) test	Hom male vs. female	p=0.8068

**SI-3 Weights**	**Main effect; F, p-value**			**Adjusted p-value**	
WT<br>♂ (n = 8, Month 3)<br>♂ (n = 9, Month 4)<br>♂ (n = 4, Month 5)<br>♂ (n = 5, Month 6)<br>♂ (n = 5, Month 7)<br>♂ (n = 5, Month 8)<br>♂ (n = 5, Month 9)<br>♂ (n = 5, Month 10)<br>♂ (n = 5, Month 11)<br>♂ (n = 5, Month 12)<br>♀ (n = 2, Month 3)<br>♀ (n = 4, Month 4)<br>♀ (n = 4, Month 5)<br>♀ (n = 4, Month 6)<br>♀ (n = 1, Month 7)<br>♀ (n = 3, Month 8)<br>♀ (n = 4, Month 9)<br>♀ (n = 4, Month 10)<br>♀ (n = 2, Month 11)<br>♀ (n = 3, Month 12)	Interaction	F(6, 50) = 0.5742;	p=0.7490	Month 4 Female vs. Male	**p=0.0172**
	Age	F(6, 50) = 7.783;	**p<0.0001**	Month 5 Female vs. Male	p=0.2502
	Sex	F (1, 50) = 22.79;	**p<0.0001**	Month 6 Female vs. Male	p=0.2502
				Month 8 Female vs. Male	p=0.5331
				Month 9 Female vs. Male	p=0.1241
				Month 10 Female vs. Male	p=0.1587
				Month 12 Female vs. Male	p=0.5963
Hemi<br>♂ (n = 1, Month 3)<br>♂ (n = 5, Month 4)<br>♂ (n = 4, Month 5)<br>♂ (n = 5, Month 6)	Interaction	F(4, 33) = 1.787;	p=0.1551	Month 4 Female vs. Male	**p=0.0306**
	Age	F(4, 33) = 12.76;	**p<0.0001**	Month 8 Female vs. Male	**p=0.0206**
	Sex	F(1, 33) = 58.58;	**p<0.0001**	Month 10 Female vs. Male	**p=0.0306**
				Month 11 Female vs. Male	**p=0.0030**

♂ (n = 5, Month 7)<br>♂ (n = 5, Month 8)<br>♂ (n = 5, Month 9)<br>♂ (n = 4, Month 10)<br>♂ (n = 4, Month 11)<br>♂ (n = 3, Month 12)<br>♀ (n = 1, Month 3)<br>♀ (n = 5, Month 4)<br>♀ (n = 1, Month 5)<br>♀ (n = 2, Month 6)<br>♀ (n = 2, Month 7)<br>♀ (n = 4, Month 8)<br>♀ (n = 1, Month 9)<br>♀ (n = 4, Month 10)<br>♀ (n = 4, Month 11)<br>♀ (n = 5, Month 12)			Month 12 Female vs. Male **p<0.0001**
Hom<br>♂ (n = 1, Month 3)<br>♂ (n = 11, Month 4)<br>♂ (n = 7, Month 5)<br>♂ (n = 8, Month 6)<br>♂ (n = 2, Month 7)<br>♀ (n = 1, Month 3)<br>♀ (n = 3, Month 4)<br>♀ (n = 3, Month 5)<br>♀ (n = 5, Month 6)<br>♀ (n = 1, Month 7)	Interaction	F(2, 31) = 1.519; p=0.2348	Month 4 Female vs. Male **p=0.0165**
	Sex	F(2, 31) = 6.890; **p=0.0033**	Month 8 Female vs. Male p=0.6113
	Genotype	F(1, 31) = 12.42; **p=0.0013**	Month 10 Female vs. Male **p=0.0165**

Supplemental Information Table SI-4. Hang time testing of mice after repetitive mild TBI (r-mTBI). Statistical analysis for combined male and female mice across hTau.P301S genotypes for Tg2541 mice. Further analysis was conducted for sex as a biological variable with 3 or more mice.

**SI-4 r-mTBI**	**Main effect; F, p-value**		**Adjusted p-value**
Hang time test, r-mTBI, WT		Log-rank (Mantel-Cox) test	Wt, r-sham vs. r-mTBI p>0.9999
r-sham<br>♂ (n = 4)<br>♀ (n = 6)		Log-rank (Mantel-Cox) test	Wt, r-sham, male vs. female p>0.9999
r-mTBI<br>♂ (n = 4)<br>♀ (n = 6)		Log-rank (Mantel-Cox) test	Wt, r-mTBI, male vs. female p>0.9999
Hang time test, r-mTBI, Hemi		Log-rank (Mantel-Cox) test	Hemi, r-sham vs. r-mTBI p=0.0533
r-sham<br>♂ (n = 8)<br>♀ (n = 13)		Log-rank (Mantel-Cox) test	Hemi, r-sham, male vs female p=0.0644
r-mTBI<br>♂ (n = 10)<br>♀ (n = 11)		Log-rank (Mantel-Cox) test	Hemi, r-mTBI, male vs female **p=0.0180**
Hang time test, r-mTBI, Hom		Log-rank (Mantel-Cox) test	Hom, r-sham vs. r-mTBI p=0.4394
r-sham<br>♂ (n = 7)<br>♀ (n = 8)		Log-rank (Mantel-Cox) test	Hom, r-sham, male vs. female p=0.0802
r-mTBI<br>♂ (n = 9)<br>♀ (n = 9)		Log-rank (Mantel-Cox) test	Hom, r-mTBI, male vs. female p=0.0647

Supplemental Information Table SI-5. Post-surgical assessments. Statistical analysis for combined male and female mice to test a main effect of injury or time (day of impact) for each hTau.P301S genotype. Further analysis was conducted for sex as a biological variable with 3 or more mice.

**SI-5 r-mTBI**	**Main effect; F, p-value**		**Adjusted p-value**
Apnea, WT	Interaction	F(4, 355) = 3.124; **p=0.0152**	Day1 r-sham vs. r-mTBI **p<0.0001**
	Injury	F(1, 355) = 479.4; **p<0.0001**	Day2 r-sham vs. r-mTBI **p<0.0001**
	Day of impact	F(4, 355) = 3.124; **p=0.0152**	Day3 r-sham vs. r-mTBI **p<0.0001**
			Day4 r-sham vs. r-mTBI **p<0.0001**
			Day5 r-sham vs. r-mTBI **p<0.0001**
r-mTBI<br>♂ (n = 22)<br>♀ (n = 15)	Interaction	F(4, 175) = 0.3919; p=0.8142	Day1 Female vs. Male p=0.8968
	Sex	F(1, 175) = 1.194; p=0.2761	Day2 Female vs. Male p=0.8968
	Day of Impact	F(4, 175) = 3.177; **p=0.0150**	Day3 Female vs. Male p=0.8968
			Day4 Female vs. Male p=0.8968
			Day5 Female vs. Male p=0.8968
Apnea, Hemi	Interaction	F(4, 613) = 5.260; **p=0.0004**	Day1 r-sham vs. r-mTBI **p<0.0001**
	Injury	F(1, 613) = 242.4; **p<0.0001**	Day2 r-sham vs. r-mTBI **p<0.0001**
	Day of impact	F(4, 613) = 5.260; **p=0.0004**	Day3 r-sham vs. r-mTBI **p<0.0001**
			Day4 r-sham vs. r-mTBI **p<0.0001**
			Day5 r-sham vs. r-mTBI **p<0.0001**
r-mTBI<br>♂ (n = 26)<br>♀ (n = 34)	Interaction	F(4, 292) = 0.4742; p=0.7547	Day1 Female vs. Male p=0.0645
	Sex	F(1, 292) = 1.194; **p=0.0015**	Day2 Female vs. Male p=0.5994
	Day of Impact	F(4, 292) = 4.314; **p=0.0021**	Day3 Female vs Male p=0.5994
			Day4 Female vs. Male p=0.3185
			Day5 Female vs. Male p=0.5994
Apnea, Hom	Interaction	F(4, 510) = 8.365; **p<0.0001**	Day1 r-sham vs. r-mTBI **p<0.0001**
	Injury	F(1, 510) = 558.5; **p<0.0001**	Day2 r-sham vs. r-mTBI **p<0.0001**
	Day of impact	F(4, 510) = 8.365; **p<0.0001**	Day3 r-sham vs. r-mTBI **p<0.0001**
			Day4 r-sham vs. r-mTBI **p<0.0001**
			Day5 r-sham vs r-mTBI **p<0.0001**
r-mTBI<br>♂ (n = 27)<br>♀ (n = 26)	Interaction	F(4, 255) = 0.6319; p=0.6401	Day1 Female vs. Male p=0.9938
	Sex	F(1, 255) = 2.843; p=0.0930	Day2 Female vs. Male p=0.9938
	Day of Impact	F(4, 255) = 8.673; **p<0.0001**	Day3 Female vs. Male p=0.8447
			Day4 Female vs. Male p=0.8711
			Day5 Female v.s Male p=0.2023
Righting reflex, WT	Interaction	F(4, 348) = 6.858; **p<0.0001**	Day1 r-sham vs. r-mTBI **p<0.0001**
	Injury	F(1, 348) = 142.1; **p<0.0001**	Day2 r-sham vs. r-mTBI **p=0.0003**
	Day of impact	F(4, 348) = 11.69; **p<0.0001**	Day3 r-sham vs. r-mTBI **p<0.0001**
			Day4 r-sham vs. r-mTBI **p=0.0001**
			Day5 r-sham vs. r-mTBI **p=0.0001**
r-sham<br>♂ (n = 18)<br>♀ (n = 18)	Interaction	F(4, 170) = 0.6829; p=0.6047	Day1 Female vs. Male p=0.3247
	Sex	F(1, 170) = 1.871; p=0.1731	Day2 Female vs. Male p=0.9701
	Day of Impact	F(4, 170) = 2.252; p=0.0655	Day3 Female vs. Male p=0.9701
			Day4 Female vs. Male p=0.7774
			Day5 Female vs. Male p=0.9701

r-mTBI<br>♂ (n = 19)<br>♀ (n = 18)	Interaction	F(4, 174) = 0.6344; p=0.6386	Day1 Female vs. Male p=0.9886
	Sex	F(1, 174) = 2.030; p=0.1560	Day2 Female vs. Male p=0.7115
	Day of Impact	F(4, 174) = 9.023; p<0.0001	Day3 Female vs. Male p=0.7633
			Day4 Female vs. Male p=0.9242
			Day5 Female vs. Male p=0.4818
Righting reflex, Hemi	Interaction	F(4, 609) = 8.175; **p<0.0001**	Day1 r-sham vs. r-mTBI **p<0.0001**
	Injury	F(1, 609) = 164.6; **p<0.0001**	Day2 r-sham vs r-mTBI **p<0.0001**
	Day of impact	F(4, 609) = 17.14; **p<0.0001**	Day3 r-sham vs. r-mTBI **p=0.0022**
			Day4 r-sham vs. r-mTBI **p<0.0001**
			Day5 r-sham vs. r-mTBI **p=0.0002**
r-sham<br>♂ (n = 29)<br>♀ (n = 35)	Interaction	F(4, 309) = 0.6319; p=0.5810	Day1 Female vs. Male p=0.8478
	Sex	F(1, 255) = 2.843; p=0.5670	Day2 Female vs. Male p=0.9274
	Day of Impact	F(4, 255) = 8.673; **p<0.0001**	Day3 Female vs. Male p=0.9274
			Day4 Female vs Male p=0.8478
			Day5 Female vs. Male p=0.8407
r-mTBI<br>♂ (n = 26)<br>♀ (n = 35)	Interaction	F(4, 295) = 1.387; p=0.2384	Day1 Female vs. Male p=0.2746
	Sex	F(1, 295) = 0.2626; p=0.6087	Day2 Female vs. Male p=0.9616
	Day of Impact	F(4, 295) = 10.71; **p<0.0001**	Day3 Female vs. Male p=0.9174
			Day4 Female vs. Male p=0.5287
			Day5 Female vs. Male p=0.9616
Righting reflex, Hom	Interaction	F(4, 508) = 13.08; **p<0.0001**	Day1 r-sham vs. r-mTBI **p<0.0001**
	Injury	F(1, 508) = 125.6; **p<0.0001**	Day2 r-sham vs.. r-mTBI **p=0.0001**
	Day of impact	F(4, 508) = 20.68; **p<0.0001**	Day3 r-sham vs. r-mTBI **p=0.0062**
			Day4 r-sham vs r-mTBI **p<0.0001**
			Day5 r-sham vs. r-mTBI **p=0.0224**
r-sham<br>♂ (n = 27)<br>♀ (n = 24)	Interaction	F(4, 244) = 1.247; p=0.2915	Day1 Female vs. Male p=0.2644
	Sex	F(1, 244) = 4.127; **p=0.0433**	Day2 Female vs. Male p=0.9545
	Day of Impact	F(4, 244) = 4.066; **p=0.0033**	Day3 Female vs. Male p=0.9832
			Day4 Female vs Male p=0.9832
			Day5 Female vs. Male p=0.0908
r-mTBI<br>♂ (n = 27)<br>♀ (n = 26)	Interaction	F(4, 255) = 0.6273; p=0.6435	Day1 Female vs. Male p=0.9392
	Sex	F(1, 255) = 0.7721; p=0.3804	Day2 Female vs. Male p=0.9398
	Day of Impact	F(4, 255) = 18.34; **p<0.0001**	Day3 Female vs. Male p=0.9392
			Day4 Female vs. Male p=0.4795
			Day5 Female vs. Male p=0.9933

**SI-5 s-TBI**	**Main effect; F, p-value**		**Adjusted p-value**
Apnea	Interaction	F(2, 251) = 0.5681; p=0.5673	WT s-sham vs. s-TBI **p<0.0001**
	Injury	F(1, 251) = 279.6; **p<0.0001**	Hemi s-sham vs. s-TBI **p<0.0001**
	Genotype	F(2, 251) = 0.5681; p=0.5673	Hom s-sham vs. s-TBI **p<0.0001**
s-TBI<br>♂ (n = 20, WT)<br>♂ (n = 23, Hemi)<br>♂ (n = 26, Hom)<br>♀ (n = 16, WT)<br>♀ (n = 21, Hemi)<br>♀ (n = 24, Hom)	Interaction	F(2, 124) = 0.4620; p=0.6311	WT Female vs. Male p=0.6120
	Sex	F(1, 124) = 4.616; **p=0.0336**	Hemi Female vs. Male p=0.1272
	Genotype	F(2, 124) = 0.6112; p=0.5443	Hom Female vs. Male p=0.6120

Righting reflex	Interaction	F(2, 267) = 1.627; p=0.1985	WT s-sham vs. s-TBI **p<0.0001**
	Injury	F(1, 267) = 627.9; **p<0.0001**	Hemi s-sham vs. s-TBI **p<0.0001**
	Genotype	F(2, 267) = 2.333; p=0.0989	Hom s-sham vs. s-TBI **p<0.0001**
s-sham<br>♂ (n = 20, WT)<br>♂ (n = 26, Hemi)<br>♂ (n = 25, Hom)<br>♀ (n = 16, WT)<br>♀ (n = 22, Hemi)<br>♀ (n = 25, Hom)	Interaction	F(2, 128) = 0.6773; p=0.5098	WT Female vs. Male p=0.9482
	Sex	F(1, 128) = 0.8687; p=0.3531	Hemi Female vs. Male p=0.9482
	Genotype	F(2, 128) = 08370; p=0.4354	Hom Female vs. Male p=0.3522
s-TBI<br>♂ (n = 26, WT)<br>♂ (n = 26, Hemi)<br>♂ (n = 30, Hom)<br>♀ (n = 14, WT)<br>♀ (n = 21, Hemi)<br>♀ (n = 30, Hom)	Interaction	F(2, 121) = 1.256; p=0.2880	WT Female vs. Male p=0.7682
	Sex	F(1, 121) = 0.03324; p=0.8556	Hemi Female vs. Male p=0.7850
	Genotype	F(2, 121) = 1.226; p=0.2967	Hom Female vs. Male p=0.3528
**s-TBI vs r-mTBI (r-mTBI day 1 only)**	**Main effect; F, p-value**		**Adjusted p-value**
Apnea	Interaction	F(2, 274) = 1.062; p=0.3470	WT s-TBI vs. s-TBI Day1 **p<0.0001**
	Injury	F(1, 274) = 111.8; **p<0.0001**	Hemi s-TBI vs. s-TBI Day1 **p<0.0001**
	Genotype	F(2, 274) = 0.01902; p=0.9812	Hom s-TBI vs. s-TBI Day1 **p<0.0001**
Righting reflex	Interaction	F(2, 283) = 0.6153; p=0.5412	WT s-TBI vs. s-TBI Day1 **p<0.0001**
	Injury	F(1, 283) = 234.3; **p<0.0001**	Hemi s-TBI vs. s-TBI Day1 **p<0.0001**
	Genotype	F(2, 283) = 2.997; p=0.0515	Hom s-TBI vs. s-TBI Day1 **p<0.0001**

Supplemental Information Table SI-6. Neuropathology of moderate single impact TBI (s-TBI). Statistical analysis for combined male and female mice to test a main effect of injury or hTau.P301S genotype for mice at each post-injury time point (1 day, 6 weeks, or 4 months). Further analysis was conducted for sex as a biological variable with 3 or more mice, with text in gray for excluded comparisons that do not have at least 3 mice of both sexes.

**SI-6 s-TBI**	**Main effect; F, p-value**			**Adjusted p-value**	
CC axon damage,<br>1 dy<br>β-APP	Interaction	F(2, 37) = 1.675;	p=0.2011	WT s-sham vs. s-TBI	**p<0.0001**
	Injury	F(1, 37) = 74.33;	**p<0.0001**	Hemi s-sham vs. s-TBI	**p=0.0006**
	Genotype	F(2, 37) = 1.675;	p=0.2012	Hom s-sham vs. s-TBI	**p<0.0001**
s-sham<br>♂ (n = 4, WT)<br>♂ (n = 4, Hemi)<br>♂ (n = 4, Hom)<br>♀ (n = 4, WT)<br>♀ (n = 4, Hemi)<br>♀ (n = 3, Hom)	Interaction	F(2, 17) = 1.521;	p=0.2468	WT Female vs. Male	p>0.9999
	Sex	F(1, 17) = 0.9638;	p=0.3400	Hemi Female vs. Male	p=0.9582
	Genotype	F(2, 17) = 1.004;	p=0.3871	Hom Female vs. Male	p=0.1709
s-TBI<br>♂ (n = 3, WT)<br>♂ (n = 4, Hemi)<br>♂ (n = 4, Hom)<br>♀ (n = 3, WT)<br>♀ (n = 4, Hemi)<br>♀ (n = 2, Hom)	Interaction	F(1, 9) = 0.1103;	p=0.7474	WT Female vs. Male	p=0.9293
	Sex	F(1, 9) = 0.0214;	p=0.8868	Hemi Female vs. Male	p=0.9293
	Genotype	F(1, 9) = 0.6838;	p=0.4297		
Cortical axon damage,<br>6 wks<br>SMI-34	Interaction	F(2, 48) = 1.154;	p=0.3241	s-sham WT vs. Hom	**p=0.0013**
	Injury	F(1, 48) = 2.148;	p=0.1493	s-sham Hemi vs. Hom	**p=0.0008**
	Genotype	F(2, 48) = 28.98;	**p<0.0001**	s-mTBI WT vs. Hom	**p<0.0001**
				s-mTBI Hemi vs. Hom	**p<0.0001**
s-sham<br>♂ (n = 3, WT)<br>♂ (n = 4, Hemi)<br>♂ (n = 4, Hom)<br>♀ (n = 5, WT)<br>♀ (n = 7, Hemi)<br>♀ (n = 4, Hom)	Interaction	F(2, 21) = 0.2308;	p=0.7959	WT Female vs. Male	p>0.9999
	Sex	F(1, 21) = 0.2350;	p=0.6328	Hemi Female vs. Male	p>0.9999
	Genotype	F(2, 21) = 11.10;	**p=0.0005**	Hom Female vs. Male	p=0.8070
s-TBI<br>♂ (n = 4, WT)<br>♂ (n = 2, Hemi)<br>♂ (n = 4, Hom)<br>♀ (n = 6, WT)<br>♀ (n = 7, Hemi)<br>♀ (n = 4, Hom)	Interaction	F(1, 14) = 0.03790;	p=0.8484	WT Female vs. Male	p>0.9999
	Sex	F(1, 14) = 0.03790;	p=0.8484	Hemi Female vs. Male	p=0.9854
	Genotype	F(1, 14) = 18.21;	**p=0.0008**		
Cortical axon damage,<br>4 mths<br>SMI-34	Interaction	F(2, 33) = 0.7357;	p=0.4869	s-sham WT vs. Hom	**p=0.0009**
	Injury	F(1, 33) = 0.4956;	p=0.4864	s-sham Hemi vs. Hom	**p=0.0001**
	Genotype	F(2, 33) = 18.45;	**p<0.0001**	s-mTBI WT vs. Hom	**p=0.0187**
				s-mTBI Hemi vs. Hom	**p=0.0171**
s-sham<br>♂ (n = 2, WT)<br>♂ (n = 5, Hemi)	Interaction	F(2, 15) = 0.6991;	p=0.5949	Hemi Female vs. Male	p>0.9999
	Sex	F(1, 15) = 0.6991;	p=0.5815	Hom Female vs. Male	p=0.4199
	Genotype	F(2, 15) = 14.0;	**p=0.0025**		

♂ (n = 5, Hom)<br>♀ (n = 2, WT)<br>♀ (n = 3, Hemi)<br>♀ (n = 4, Hom)			
s-TBI<br>♂ (n = 3, WT)<br>♂ (n = 4, Hemi)<br>♂ (n = 3, Hom)<br>♀ (n = 2, WT)<br>♀ (n = 3, Hemi)<br>♀ (n = 4, Hom)	Interaction<br>Sex<br>Genotype	F(1, 10) = 6.286; **p=0.0311**<br>F(1, 10) = 2.790; p=0.1188<br>F(1, 10) = 20.70; **p=0.0011**	Hemi Female vs. Male p=0.7521<br>Hom Female vs. Male p=0.0182
CC width,<br>6 wks<br>H & E	Interaction<br>Injury<br>Genotype	F(2, 26) = 2.234; p=0.1273<br>F(1, 26) = 18.05; **p=0.0002**<br>F(2, 26) = 2.431; p=0.1077	WT s-sham vs. s-TBI **p=0.0017**<br>Hemi s-sham vs. s-TBI p=0.0648<br>Hom s-sham vs. s-TBI p=0.2714
s-sham<br>♂ (n = 3, WT)<br>♂ (n = 1, Hemi)<br>♂ (n = 1, Hom)<br>♀ (n = 2, WT)<br>♀ (n = 5, Hemi)<br>♀ (n = 4, Hom)			
s-TBI<br>♂ (n = 4, WT)<br>♂ (n = 5, Hemi)<br>♂ (n = 3, Hom)<br>♀ (n = 0, WT)<br>♀ (n = 2, Hemi)<br>♀ (n = 3, Hom)		t-test	Hom Female vs. Male **p=0.0394**
CC width,<br>4 mths<br>H & E	Interaction<br>Injury<br>Genotype	F(2, 30) = 0.0770; p=0.9261<br>F(1, 30) = 21.61; **p<0.0001**<br>F(2, 30) = 1.456; p=0.2492	WT s-sham vs. s-TBI **p=0.0252**<br>Hemi s-sham vs. s-TBI **p=0.0237**<br>Hom s-sham vs. s-TBI **p=0.0252**
s-sham<br>♂ (n = 3, WT)<br>♂ (n = 4, Hemi)<br>♂ (n = 3, Hom)<br>♀ (n = 3, WT)<br>♀ (n = 2, Hemi)<br>♀ (n = 2, Hom)		t-test	WT Female vs. Male p=0.6501
s-TBI<br>♂ (n = 4, WT)<br>♂ (n = 3, Hemi)<br>♂ (n = 1, Hom)<br>♀ (n = 4, WT)<br>♀ (n = 3, Hemi)<br>♀ (n = 4, Hom)	Interaction<br>Sex<br>Genotype	F(1, 10) = 0.0021; p=0.9645<br>F(1, 10) = 0.2172; p=0.6512<br>F(1, 10) = 0.8742; p=0.3718	WT Female vs. Male p=0.9125<br>Hemi Female vs. Male p=0.9125
CC width,<br>4 mths<br>MBP	Interaction<br>Injury<br>Genotype	F(2, 28) = 0.04798; p=0.9532<br>F(1, 28) = 27.99; **p<0.0001**<br>F(2, 28) = 2.749; p=0.0813	WT s-sham vs. s-TBI **p=0.0071**<br>Hemi s-sham vs. s-TBI **p=0.0098**<br>Hom s-sham vs. s-TBI **p=0.0098**
s-sham<br>♂ (n = 3, WT)		t-test	WT Female vs. Male p=0.4021

♂ (n = 2, Hemi)<br>♂ (n = 3, Hom)<br>♀ (n = 3, WT)<br>♀ (n = 0, Hemi)<br>♀ (n = 2, Hom)			
s-TBI<br>♂ (n = 3, WT)<br>♂ (n = 3, Hemi)<br>♂ (n = 2, Hom)<br>♀ (n = 4, WT)<br>♀ (n = 3, Hemi)<br>♀ (n = 4, Hom)	Interaction	F(1, 9) = 0.4379; p=0.5247	WT Female vs. Male p=0.9039
	Sex	F(1, 9) = 0.6911; p=0.4273	Hemi Female vs Male p=0.5556
	Genotype	F(1, 9) = 0.5776; p=0.4667	
CC myelin,<br>6 wks<br>MBP	Interaction	F(2, 29) = 0.1316; p=0.8772	WT s-sham vs. s-TBI p=0.9995
	Injury	F(1, 29) = 0.2774; p=0.6024	Hemi s-sham vs. s-TBI p=0.9996
	Genotype	F(2, 29) = 0.9195; p=0.4100	Hom s-sham vs. s-TBI p=0.9996
s-sham<br>♂ (n = 3, WT)<br>♂ (n = 3, Hemi)<br>♂ (n = 2, Hom)<br>♀ (n = 3, WT)<br>♀ (n = 2, Hemi)<br>♀ (n = 2, Hom)		t-test	WT Female vs. Male p=0.5534
s-TBI<br>♂ (n = 5, WT)<br>♂ (n = 4, Hemi)<br>♂ (n = 2, Hom)<br>♀ (n = 4, WT)<br>♀ (n = 3, Hemi)<br>♀ (n = 2, Hom)	Interaction	F(2, 12) = 2.456; p=0.1431	WT Female vs. Male p=0.3948
	Sex	F(1, 12) = 0.01758; p=0.8967	Hemi Female vs. Male p=0.3948
	Genotype	F(2, 12) = 0.4823; p=0.5006	
CC myelin,<br>4 mths<br>MBP	Interaction	F(2, 26) = 1.980; p=0.1583	Hom s-sham vs. s-TBI **p=0.0107**
	Injury	F(1, 26) = 11.44; **p=0.0023**	s-sham Wt vs. Hom **p=0.0132**
	Genotype	F(2, 26) = 23.19; **p<0.0001**	s-sham Hemi vs. Hom **p=0.0132**
			s-TBI Wt vs. Hom **p<0.0001**
			s-TBI Hemi vs. Hom **p<0.0001**
s-sham<br>♂ (n = 4, WT)<br>♂ (n = 3, Hemi)<br>♂ (n = 3, Hom)<br>♀ (n = 3, WT)<br>♀ (n = 1, Hemi)<br>♀ (n = 1, Hom)		t-test	WT Female vs. Male p=0.0846
s-TBI<br>♂ (n = 3, WT)<br>♂ (n = 5, Hemi)<br>♂ (n = 1, Hom)<br>♀ (n = 3, WT)<br>♀ (n = 1, Hemi)<br>♀ (n = 3, Hom)		t-test	WT Female vs. Male p=0.7601

Cortical myelin,<br>6 wks<br>MBP	Interaction	F (2, 25) = 0.4838; p=0.6221	s-sham Wt vs. Hemi p=0.5269
	Injury	F (1, 25) = 1.011; p=0.3242	s-sham Wt vs. Hom p=0.0823
	Genotype	F (2, 25) = 3.809; **p=0.0360**	s-sham Hemi vs. Hom p=0.1593
			s-TBI Wt vs. Hemi p=0.4991
			s-TBI Wt vs. Hom p=0.3614
			s-TBI Hemi vs. Hom p=0.6288
s-sham<br>♂ (n = 3, WT)<br>♂ (n = 4, Hemi)<br>♂ (n = 3, Hom)<br>♀ (n = 3, WT)<br>♀ (n = 2, Hemi)<br>♀ (n = 1, Hom)		t-test	WT Female vs. Male p=0.1836
s-TBI<br>♂ (n = 5, WT)<br>♂ (n = 3, Hemi)<br>♂ (n = 2, Hom)<br>♀ (n = 2, WT)<br>♀ (n = 2, Hemi)<br>♀ (n = 2, Hom)			
Cortical myelin,<br>4 mths<br>MBP	Interaction	F(2, 24) = 0.2920; p=0.7494	s-sham Wt vs. Hemi p=0.8920
	Injury	F(1, 21) = 3.402; p=0.0775	s-sham Wt vs. Hom **p=0.0004**
	Genotype	F(2, 21) = 22.57; **p<0.0001**	s-sham Hemi vs. Hom **p=0.0003**
			s-TBI Wt vs. Hemi p=0.7695
			s-TBI Wt vs. Hom **p=0.0026**
			s-TBI Hemi vs. Hom **p=0.0026**
s-sham<br>♂ (n = 2, WT)<br>♂ (n = 3, Hemi)<br>♂ (n = 3, Hom)<br>♀ (n = 3, WT)<br>♀ (n = 3, Hemi)<br>♀ (n = 1, Hom)		t-test	Hemi Female vs. Male p=0.4901
s-TBI<br>♂ (n = 4, WT)<br>♂ (n = 3, Hemi)<br>♂ (n = 2, Hom)<br>♀ (n = 2, WT)<br>♀ (n = 1, Hemi)<br>♀ (n = 3, Hom)			
CC microglia<br>6 wks<br>IBA1	Interaction	F(2, 38) = 2.025; p=0.1460	WT s-sham vs. s-TBI p=0.3277
	Injury	F(1, 38) = 15.19; **p=0.0004**	Hemi s-sham vs. s-TBI p=0.1164
	Genotype	F(2, 38) = 3.069; p=0.0581	Hom s-sham vs. s-TBI **p=0.0009**
s-sham<br>♂ (n = 4, WT)<br>♂ (n = 3, Hemi)<br>♂ (n = 3, Hom)<br>♀ (n = 4, WT)	Interaction	F(2, 15) = 1.469; p=0.2633	WT Female vs. Male p=0.8462
	Sex	F(1, 15) = 0.07338; p=0.7902	Hemi Female vs. Male p=0.6985
	Genotype	F(2, 15) = 1.204; p=0.3273	Hom Female vs. Male p=0.3593

♀ (n = 3, Hemi)<br>♀ (n = 5, Hom)			
s-TBI<br>♂ (n = 4, WT)<br>♂ (n = 2, Hemi)<br>♂ (n = 5, Hom)<br>♀ (n = 4, WT)<br>♀ (n = 4, Hemi)<br>♀ (n = 4, Hom)	Interaction<br>Sex<br>Genotype	F(1, 13) = 26.95; **p=0.0002**<br>F(1, 13) = 21.40; **p=0.0005**<br>F(1, 13) = 29.92; **p=0.0001**	WT Female vs. Male p=0.7033<br>Hom Female vs. Male **p<0.0001**
CC microglia<br>4 mths<br>IBA1	Interaction<br>Injury<br>Genotype	F(2, 41) = 3.256; **p=0.0487**<br>F(1, 41) = 20.87; **p<0.0001**<br>F(2, 41) = 3.004; p=0.0606	WT s-sham vs. s-TBI p=0.4232<br>Hemi s-sham vs. s-TBI **p=0.0218**<br>Hom s-sham vs. s-TBI **p=0.0002**
s-sham<br>♂ (n = 3, WT)<br>♂ (n = 4, Hemi)<br>♂ (n = 5, Hom)<br>♀ (n = 5, WT)<br>♀ (n = 3, Hemi)<br>♀ (n = 4, Hom)	Interaction<br>Sex<br>Genotype	F(2, 17) = 0.5559; p=0.5836<br>F(1, 17) = 2.395; p=0.1401<br>F(2, 17) = 0.2672; p=0.7686	WT Female vs. Male p=0.3096<br>Hemi Female vs. Male p=0.8585<br>Hom Female vs. Male p=0.6532
s-TBI<br>♂ (n = 4, WT)<br>♂ (n = 5, Hemi)<br>♂ (n = 4, Hom)<br>♀ (n = 4, WT)<br>♀ (n = 4, Hemi)<br>♀ (n = 3, Hom)	Interaction<br>Sex<br>Genotype	F(2, 18) = 3.238; p=0.0629<br>F(1, 18) = 3.009; p=0.0999<br>F(2, 18) = 3.511; p=0.0516	WT Female vs. Male p=0.5164<br>Hemi Female vs. Male p=0.5164<br>Hom Female vs. Male p=0.0530
Cortical microglia<br>6 wks<br>IBA1	Interaction<br>Injury<br>Genotype	F(2, 36) = 1.071; p=0.3532<br>F(1, 36) = 2.447; p=0.1265<br>F(2, 36) = 2.828; p=0.0723	WT s-sham vs. s-TBI p>0.9823<br>Hemi s-sham vs. s-TBI p>0.9160<br>Hom s-sham vs. s-TBI p>0.6672
s-sham<br>♂ (n = 4, WT)<br>♂ (n = 3, Hemi)<br>♂ (n = 3, Hom)<br>♀ (n = 4, WT)<br>♀ (n = 3, Hemi)<br>♀ (n = 3, Hom)	Interaction<br>Sex<br>Genotype	F(2, 13) = 1.988; p=0.1764<br>F(1, 13) = 0.03049; p=0.8641<br>F(2, 13) = 0.5043; p=0.6153	WT Female vs. Male p=0.7567<br>Hemi Female vs. Male p=0.4592<br>Hom Female vs. Male p=0.4592
s-TBI<br>♂ (n = 4, WT)<br>♂ (n = 2, Hemi)<br>♂ (n = 5, Hom)<br>♀ (n = 4, WT)<br>♀ (n = 4, Hemi)<br>♀ (n = 4, Hom)	Interaction<br>Sex<br>Genotype	F(1, 13) = 20.14; **p=0.0006**<br>F(1, 13) = 18.95; **p=0.0008**<br>F(1, 13) = 37.57; **p<0.0001**	WT Female vs. Male p=0.9274<br>Hom Female vs. Male **p<0.0001**
Cortical microglia<br>4 months<br>IBA1	Interaction<br>Injury<br>Genotype	F(2, 41) = 0.5112; p=0.6036<br>F(1, 41) = 0.0003; p=0.9872<br>F(2, 41) =4.400; **p=0.0186**	s-TBI WT vs. Hemi **p=0.0213**<br>s-TBI WT vs. Hom p=0.2624<br>s-TBI Hemi vs. Hom p=0.2624
s-sham<br>♂ (n = 3, WT)<br>♂ (n = 4, Hemi)<br>♂ (n = 5, Hom)<br>♀ (n = 4, WT)	Interaction<br>Sex<br>Genotype	F(2, 17) = 0.1511; p=0.8609<br>F(1, 17) = 3.223; p=0.0904<br>F(2, 17) = 1.836; p=0.1897	WT Female vs. Male p=0.4921<br>Hemi Female vs. Male p=0.5848<br>Hom Female vs. Male p=0.4921

♀ (n = 3, Hemi)<br>♀ (n = 4, Hom)			
s-TBI<br>♂ (n = 4, WT)<br>♂ (n = 5, Hemi)<br>♂ (n = 3, Hom)<br>♀ (n = 4, WT)<br>♀ (n = 4, Hemi)<br>♀ (n = 4, Hom)	Interaction	F(2, 18) = 0.5417; p=0.5910	WT Female vs. Male p=0.9349
	Sex	F(1, 18) = 0.2708; p=0.6091	Hemi Female vs. Male p=0.5979
	Genotype	F(2, 18) = 3.102; p=0.0696	Hom Female vs. Male p=0.9349
CC astrocytes<br>6 wks<br>GFAP	Interaction	F(2, 39) = 0.2175; p=0.8055	WT s-sham vs. s-TBI p=0.2426
	Injury	F(2, 39) = 8.477; **p=0.0059**	Hemi s-sham vs. s-TBI p=0.1562
	Genotype	F(2, 39) = 1.863; p=0.1688	Hom s-sham vs. s-TBI p=0.1322
s-sham<br>♂ (n = 5, WT)<br>♂ (n = 3, Hemi)<br>♂ (n = 5, Hom)<br>♀ (n = 2, WT)<br>♀ (n = 5, Hemi)<br>♀ (n = 4, Hom)	Interaction	F(1, 13) = 4.922; **p=0.0449**	Hemi Female vs. Male **p=0.0151**
	Sex	F(1, 13) = 5.915; **p=0.0302**	Hom Female vs. Male p=0.8770
	Genotype	F(1, 13) = 7.995; **p=0.0143**	
s-TBI<br>♂ (n = 6, WT)<br>♂ (n = 3, Hemi)<br>♂ (n = 4, Hom)<br>♀ (n = 2, WT)<br>♀ (n = 4, Hemi)<br>♀ (n = 5, Hom)	Interaction	F(1, 12) = 0.03668; p=0.8513	Hemi Female vs. Male p=0.3983
	Sex	F(1, 12) = 3.006; p=0.1086	Hom Female v.s Male p=0.3983
	Genotype	F(1, 12) = 6.902; p=0.0221	
CC astrocytes<br>4 mths<br>GFAP	Interaction	F(2, 41) = 3.285; **p=0.0475**	Wt s-sham vs. s-TBI p=0.1961
	Injury	F(1, 41) = 25.79; **p<0.0001**	Hemi s-sham vs. s-TBI **p<0.0001**
	Genotype	F(2, 41) =11.42; **p=0.0001**	Hom s-sham vs. s-TBI **p=0.0320**
s-sham<br>♂ (n = 3, WT)<br>♂ (n = 3, Hemi)<br>♂ (n = 5, Hom)<br>♀ (n = 4, WT)<br>♀ (n = 4, Hemi)<br>♀ (n = 3, Hom)	Interaction	F(2, 18) = 0.213; p=0.8098	WT Female vs. Male p=0.9472
	Sex	F(1, 18) = 0.006; p=0.9366	Hemi Female vs. Male p=0.9472
	Genotype	F(2, 18) = 13.07; **p=0.0003**	Hom Female vs. Male p=0.9472
s-TBI<br>♂ (n = 3, WT)<br>♂ (n = 5, Hemi)<br>♂ (n = 3, Hom)<br>♀ (n = 4, WT)<br>♀ (n = 4, Hemi)<br>♀ (n = 4, Hom)	Interaction	F(2, 17) = 3.284; p=0.0622	WT Female vs. Male p=0.1146
	Sex	F(1, 17) = 0.0988; p=0.7571	Hemi Female vs. Male p=0.5681
	Genotype	F(2, 17) = 7.619; **p=0.0043**	Hom Female vs. Male p=0.5681
Cortical astrocytes<br>6 wks<br>GFAP	Interaction	F(2, 38) = 0.1002; p=0.9049	s-sham WT vs. Hemi p=0.4812
	Injury	F(1, 38) = 2.871; p=0.0984	s-sham Hemi vs. Hom p=0.7138
	Genotype	F(2, 38) = 3.578; **p=0.0377**	s-TBI WT vs. Hemi p=0.3376
s-sham<br>♂ (n = 4, WT)<br>♂ (n = 3, Hemi)<br>♂ (n = 3, Hom)<br>♀ (n = 2, WT)<br>♀ (n = 6, Hemi)	Interaction	F(1, 12) = 0.8745; p=0.3682	Hemi Female vs. Male p=0.5254
	Sex	F(1, 12) = 0.2518; p=0.6249	Hom Female vs. Male p=0.7728
	Genotype	F(1, 12) = 3.123; p=0.1026	

♀ (n = 4, Hom)			
s-TBI<br>♂ (n = 4, WT)<br>♂ (n = 3, Hemi)<br>♂ (n = 3, Hom)<br>♀ (n = 4, WT)<br>♀ (n = 4, Hemi)<br>♀ (n = 4, Hom)	Interaction	F(2, 16) = 0.05077; p=0.9506	WT Female vs. Male p=0.8468
	Sex	F(1, 16) = 0.6778; p=0.6778	Hemi Female vs. Male p=0.8911
	Genotype	F(2, 16) = 1.308; p=0.2977	Hom Female vs. Male p=0.8911
Cortical astrocytes<br>4 mths<br>GFAP	Interaction	F(2, 39) = 0.5374; p=0.5885	s-sham WT vs. Hemi p=0.4494
	Injury	F(1, 39) = 2.151; p=0.1505	s-sham Hemi vs. Hom p=0.4976
	Genotype	F(2, 39) = 5.452; **p=0.0082**	s-TBI WT vs. Hemi **p=0.0318**
s-sham<br>♂ (n = 3, WT)<br>♂ (n = 5, Hemi)<br>♂ (n = 5, Hom)<br>♀ (n = 4, WT)<br>♀ (n = 4, Hemi)<br>♀ (n = 3, Hom)	Interaction	F(2, 18) = 1.413; p=0.2691	WT Female vs. Male p=0.4201
	Sex	F(1, 18) = 0.2874; p=0.5985	Hemi Female vs. Male p=0.6135
	Genotype	F(2, 18) = 2.229; p=0.1365	Hom Female vs. Male p=0.8143
s-TBI<br>♂ (n = 3, WT)<br>♂ (n = 4, Hemi)<br>♂ (n = 3, Hom)<br>♀ (n = 4, WT)<br>♀ (n = 5, Hemi)<br>♀ (n = 4, Hom)	Interaction	F(2, 17) = 0.6078; p=0.5560	WT Female vs. Male p=0.4672
	Sex	F(1, 17) = 0.0988; p=0.3885	Hemi Female vs. Male p=0.9700
	Genotype	F(2, 17) = 7.619; p=0.0896	Hom Female vs. Male p=0.9700

Supplemental Information Table SI-7. Neuropathology of repetitive mild TBI (r-mTBI).
Statistical analysis for combined male and female mice to test a main effect of injury or hTau.P301S genotype for mice at each post-injury time point (1 day, 6 weeks, or 4 months). Further analysis was conducted for sex as a biological variable with 3 or more mice, with text in gray for excluded comparisons that do not have at least 3 mices of both sexes.

SI-7 r-mTBI	Main effect; F, p-value			Adjusted p-value	
CC axon damage<br>1 dy<br>βAPP	Interaction	F(2, 34) = 5.466;	**p=0.0087**	WT r-sham vs. r-mTBI	**p<0.0001**
	Injury	F(1, 34) = 23.06;	**p<0.0001**	r-mTBI WT vs. Hom	**p=0.0005**
	Genotype	F(2, 34) = 6.147;	**p=0.0053**	r-mTBI WT vs. Hom	**p=0.0021**
r-sham<br>♂ (n = 3, WT)<br>♂ (n = 3, Hemi)<br>♂ (n = 4, Hom)<br>♀ (n = 4, WT)<br>♀ (n = 4, Hemi)<br>♀ (n = 3, Hom)	Interaction	F(2, 16) = 0.294;	p=0.7490	WT Female vs. Male	p=0.7237
	Sex	F(1, 16) = 0.279;	p=0.6042	Hemi Female vs. Male	p>0.9999
	Genotype	F(2, 16) = 1.390;	p=0.2776	Hom Female vs. Male	p>0.9999
r-mTBI<br>♂ (n = 4, WT)<br>♂ (n = 3, Hemi)<br>♂ (n = 3, Hom)<br>♀ (n = 4, WT)<br>♀ (n = 3, Hemi)<br>♀ (n = 2, Hom)	Interaction	F(1, 9) = 0.06024;	p=0.8116	WT Female vs. Male	p=0.8043
	Sex	F(1, 9) = 0.3440;	p=0.5719	Hemi Female vs. Male	p=0.8206
	Genotype	F(1, 9) = 5.377;	**p=0.0456**		
Cortical axon damage,<br>6 wks<br>SMI-34	Interaction	F(2, 40) = 4.238;	**p=0.0214**	Hom r-sham vs. r-mTBI	**p=0.0002**
	Injury	F(1, 40) = 12.77;	**p=0.0009**	r-mTBI WT vs. Hom	**p=0.0007**
	Genotype	F(2, 40) =7.951;	**p=0.0012**	r-mTBI Hemi vs. Hom	**p=0.0005**
r-sham<br>♂ (n = 3, WT)<br>♂ (n = 3, Hemi)<br>♂ (n = 9, Hom)<br>♀ (n = 4, WT)<br>♀ (n = 4, Hemi)<br>♀ (n = 7, Hom)	Interaction	F(2, 24) = 1.094;	p=0.3511	WT Female vs. Male	p>0.9999
	Sex	F(1, 24) = 1.147;	p=0.2948	Hemi Female vs. Male	p=0.9835
	Genotype	F(2, 24) = 3.571;	**p=0.0439**	Hom Female vs. Male	p=0.0914
r-mTBI<br>♂ (n = 2, WT)<br>♂ (n = 5, Hemi)<br>♂ (n = 4, Hom)<br>♀ (n = 5, WT)<br>♀ (n = 3, Hemi)<br>♀ (n = 5, Hom)	Interaction	F(1, 13) = 0.1006;	p=0.7562	Hemi Female vs. Male	p=0.9428
	Sex	F(1, 13) = 0.04382;	p=0.8374	Hom Female vs. Male	p=0.9121
	Genotype	F(1, 13) = 6.919;	**p=0.0208**		
Cortical axon damage,<br>4 mths<br>SMI-34	Interaction	F(2, 37) = 0.8010;	p=0.4565	r-sham; WT vs. Hom	p=0.0628
	Injury	F(1, 37) = 0.5931;	p=0.4461	r-sham; Hemi vs. Hom	p=0.0628
	Genotype	F(2, 37) = 14.23;	**p<0.0001**	r-mTBI; WT vs. Hom	**p=0.0009**
r-sham<br>♂ (n = 3, WT)<br>♂ (n = 3, Hemi)<br>♂ (n = 4, Hom)<br>♀ (n = 3, WT)<br>♀ (n = 5, Hemi)	Interaction	F(2, 15) = 1.884;	p=0.1863	WT Female vs. Male	p>0.9999
	Sex	F(1, 15) = 1.255;	p=0.1255	Hemi Female vs. Male	p=0.9412
	Genotype	F(2, 15) = 10.54;	**p=0.0014**	Hom Female vs. Male	p=0.0668

♀ (n = 3, Hom)			
r-mTBI<br>♂ (n = 3, WT)<br>♂ (n = 2, Hemi)<br>♂ (n = 5, Hom)<br>♀ (n = 2, WT)<br>♀ (n = 5, Hemi)<br>♀ (n = 5, Hom)		t-test	Hom Female vs. Male p=0.8283
CC width,<br>6 wks<br>H & E	Interaction	F(2, 30) = 0.0051; p=0.9949	WT r-sham vs. r-mTBI p=0.9998
	Injury	F(1, 30) = 0.3080; p=0.5830	Hemi r-sham vs. r-mTBI p=0.9998
	Genotype	F(2, 30) = 0.7202; p=0.4949	Hom r-sham vs. r-mTBI p=0.9998
r-sham<br>♂ (n = 2, WT)<br>♂ (n = 3, Hemi)<br>♂ (n = 1, Hom)<br>♀ (n = 3, WT)<br>♀ (n = 3, Hemi)<br>♀ (n = 5, Hom)		t-test	Hemi Female vs. Male p=0.9716
r-mTBI<br>♂ (n = 3, WT)<br>♂ (n = 5, Hemi)<br>♂ (n = 3, Hom)<br>♀ (n = 3, WT)<br>♀ (n = 5, Hemi)<br>♀ (n = 3, Hom)	Interaction	F(2, 16) = 1.592; p=0.2341	WT Female vs. Male p=0.9960
	Sex	F(1, 16) = 1.684; p=0.2127	Hemi Female vs. Male p=0.9960
	Genotype	F(2, 16) = 0.4564; p=0.6416	Hom Female vs. Male p=0.1537
CC width,<br>4 mths<br>H & E	Interaction	F(2, 29) = 2.521; p=0.0978	r-mTBI WT vs. Hom **p=0.0034**
	Injury	F(1, 29) = 11.61; **p=0.0019**	r-mTBI Hemi vs. Hom **p=0.0076**
	Genotype	F(2, 29) = 7.647 **p=0.0022**	Hom r-sham vs. r-mTBI **p=0.0022**
r-sham<br>♂ (n = 3, WT)<br>♂ (n = 4, Hemi)<br>♂ (n = 3, Hom)<br>♀ (n = 2, WT)<br>♀ (n = 1, Hemi)<br>♀ (n = 3, Hom)		t-test	Hom Female vs. Male p=0.9024
r-mTBI<br>♂ (n = 3, WT)<br>♂ (n = 5, Hemi)<br>♂ (n = 3, Hom)<br>♀ (n = 3, WT)<br>♀ (n = 5, Hemi)<br>♀ (n = 3, Hom)	Interaction	F(2, 13) = 1.9207; p=0.4227	WT Female vs. Male p=0.7550
	Sex	F(1, 13) = 0.001261; p=0.9722	Hemi Female vs. Male p=0.6642
	Genotype	F(2, 13) = 6.604; p=0.0105	Hom Female vs. Male p=0.7550
CC width,<br>4 mths<br>MBP	Interaction	F(2, 28) = 2.201; p=0.1295	r-mTBI WT vs. Hom **p=0.0154**
	Injury	F(1, 28) = 6.182; **p=0.0191**	r-mTBI Hemi vs. Hom **p=0.0029**
	Genotype	F(2, 28) = 6.481 **p=0.0049**	Hom r-sham vs. r-mTBI **p=0.0224**
r-sham<br>♂ (n = 3, WT)<br>♂ (n = 2, Hemi)<br>♂ (n = 3, Hom)<br>♀ (n = 3, WT)		t-test	WT Female vs. Male p=0.7322

♀ (n = 3, Hemi)<br>♀ (n = 2, Hom)			
r-mTBI<br>♂ (n = 3, WT)<br>♂ (n = 3, Hemi)<br>♂ (n = 4, Hom)<br>♀ (n = 2, WT)<br>♀ (n = 2, Hemi)<br>♀ (n = 4, Hom)		t-test	Hom Female vs. Male p=0.2669
CC myelin,<br>6 wks<br>MBP	Interaction	F(2, 20) = 0.7721; p=0.4753	r-mTBI Wt vs. Hemi p=0.0526
	Injury	F(1, 20) = 1.529; p=0.2306	r-mTBI Wt vs. Hom p=0.5391
	Genotype	F(2, 20) = 4.138; **p=0.0313**	r-mTBI Hemi vs. Hom p=0.0694
r-sham<br>♂ (n = 2, WT)<br>♂ (n = 3, Hemi)<br>♂ (n = 5, Hom)<br>♀ (n = 2, WT)<br>♀ (n = 1, Hemi)<br>♀ (n = 1, Hom)			
r-mTBI<br>♂ (n = 3, WT)<br>♂ (n = 4, Hemi)<br>♂ (n = 4, Hom)<br>♀ (n = 0, WT)<br>♀ (n = 0, Hemi)<br>♀ (n = 1, Hom)			
CC myelin,<br>4 mths<br>MBP	Interaction	F(2, 28) = 0.8503; p=0.4380	Wt s-sham vs. s-TBI p=0.7310
	Injury	F(1, 28) = 0.02167; p=0.8840	Hemi s-sham vs. s-TBI p=0.7310
	Genotype	F(2, 28) = 0.6013; p=0.5550	Hom s-sham vs. s-TBI p=0.7310
r-sham<br>♂ (n = 3, WT)<br>♂ (n = 3, Hemi)<br>♂ (n = 5, Hom)<br>♀ (n = 2, WT)<br>♀ (n = 2, Hemi)<br>♀ (n = 0, Hom)			
r-mTBI<br>♂ (n = 4, WT)<br>♂ (n = 2, Hemi)<br>♂ (n = 3, Hom)<br>♀ (n = 3, WT)<br>♀ (n = 4, Hemi)<br>♀ (n = 3, Hom)	Interaction	F(1, 9) = 2.468; p=0.1506	WT Female vs. Male p=0.2232
	Sex	F(1, 9) = 0.6171; p=0.4523	Hom Female vs. Male p=0.6038
	Genotype	F(1, 9) = 0.002995; p=0.9576	
Cortical myelin,<br>6 wks<br>MBP	Interaction	F(2, 21) = 0.0316; p=0.9689	Wt r-sham vs. r-mTBI p=0.4158
	Injury	F(1, 21) = 4.684; **p=0.0421**	Hemi r-sham vs. r-mTBI p=0.4158
	Genotype	F(2, 21) = 0.4957; p=0.6161	Hom r-sham vs. r-mTBI p=0.4158
r-sham<br>♂ (n = 1, WT)<br>♂ (n = 2, Hemi)			

♂ (n = 2, Hom)<br>♀ (n = 2, WT)<br>♀ (n = 1, Hemi)<br>♀ (n = 2, Hom)			
r-mTBI<br>♂ (n = 4, WT)<br>♂ (n = 5, Hemi)<br>♂ (n = 4, Hom)<br>♀ (n = 2, WT)<br>♀ (n = 2, Hemi)<br>♀ (n = 2, Hom)			
Cortical myelin,<br>4 mths<br>MBP	Interaction	F (2, 21) = 1.021; p=0.3775	Wt s-sham vs. s-TBI p=0.2377
	Injury	F (1, 21) = 2.034; p=0.1685	Hemi s-sham vs. s-TBI p=0.8957
	Genotype	F (2, 21) = 0.1011; p=0.9043	Hom s-sham vs. s-TBI p=0.9481
r-sham<br>♂ (n = 3, WT)<br>♂ (n = 2, Hemi)<br>♂ (n = 4, Hom)<br>♀ (n = 1, WT)<br>♀ (n = 2, Hemi)<br>♀ (n = 2, Hom)			
r-mTBI<br>♂ (n = 2, WT)<br>♂ (n = 1, Hemi)<br>♂ (n = 5, Hom)<br>♀ (n = 1, WT)<br>♀ (n = 2, Hemi)<br>♀ (n = 2, Hom)			
CC microglia<br>6 wks<br>IBA1	Interaction	F(2, 42) = 1.063; p=0.3545	WT r-sham vs. r-mTBI p=0.3854
	Injury	F(1, 42) = 10.83; **p=0.0020**	Hemi r-sham vs. r-mTBI p=0.1340
	Genotype	F(2, 42) = 2.340; p=0.1088	Hom r-sham vs. r-mTBI **p=0.0137**
r-sham<br>♂ (n = 3, WT)<br>♂ (n = 2, Hemi)<br>♂ (n = 5, Hom)<br>♀ (n = 5, WT)<br>♀ (n = 4, Hemi)<br>♀ (n = 3, Hom)	Interaction	F(1, 12) = 6.616; **p=0.0299**	WT Female vs. Male p=0.1438
	Sex	F(1, 12) = 0.0354 p=0.8539	Hom Female vs. Male p=0.1438
	Genotype	F(2, 12) = 1.010; p=0.3347	
r-mTBI<br>♂ (n = 4, WT)<br>♂ (n = 4, Hemi)<br>♂ (n = 5, Hom)<br>♀ (n = 4, WT)<br>♀ (n = 5, Hemi)<br>♀ (n = 5, Hom)	Interaction	F(2, 20) = 0.7593; p=0.1730	WT Female vs. Male p=0.8183
	Sex	F(1, 20) = 4.186; p=0.1724	Hemi Female vs. Male p=0.8183
	Genotype	F(2, 20) = 8.157; p=0.0925	Hom Female vs. Male p=0.0906
CC microglia<br>4 mths<br>IBA1	Interaction	F(2, 40) = 4.238; **p=0.0214**	Hom r-sham vs. r-mTBI **p=0.0002**
	Injury	F(1, 40) = 12.77; **p=0.0009**	r-mTBI WT vs. Hom **p=0.0007**
	Genotype	F(2, 40) =7.951; **p=0.0012**	r-mTBI Hemi vs. Hom **p=0.0005**
r-sham<br>♂ (n = 3, WT)	Interaction	F(2, 16) = 0.1819; p=0.8354	WT Female vs. Male p=0.8093
	Sex	F(1, 16) = 0.4278; p=0.5224	Hemi Female vs. Male p=0.9735

♂ (n = 5, Hemi)<br>♂ (n = 4, Hom)<br>♀ (n = 3, WT)<br>♀ (n = 4, Hemi)<br>♀ (n = 3, Hom)	Genotype	F(2, 16) = 3.986; **p=0.0394**	Hom Female vs. Male p=0.9735
r-mTBI<br>♂ (n = 3, WT)<br>♂ (n = 3, Hemi)<br>♂ (n = 5, Hom)<br>♀ (n = 2, WT)<br>♀ (n = 6, Hemi)<br>♀ (n = 5, Hom)	Interaction	F(1, 15) < 0.0001; p=0.9988	Hemi Female vs. Male p=0.7225
	Sex	F(1, 15) = 0.9591; p=0.3429	Hom Female vs. Male p=0.7225
	Genotype	F(1, 15) = 9.696; **p=0.0071**	
Cortex microglia<br>6 wks<br>IBA1	Interaction	F(2, 41) = 0.3493; p=0.7073	r-sham WT vs. Hom p=0.1588
	Injury	F(1, 41) = 0.4082; p=0.5264	r-mTBI WT vs. Hom p=0.2703
	Genotype	F(2, 41) = 3.451; **p=0.0412**	r-mTBI Hemi vs. Hom p=0.1815
r-sham<br>♂ (n = 3, WT)<br>♂ (n = 2, Hemi)<br>♂ (n = 5, Hom)<br>♀ (n = 5, WT)<br>♀ (n = 4, Hemi)<br>♀ (n = 3, Hom)	Interaction	F(1, 11) = 0.8250; p=0.3832	WT Female vs. Male p=0.7052
	Sex	F(1, 11) = 0.0247; p=0.8780	Hom Female vs. Male p=0.7052
	Genotype	F(1, 11) = 3.773; p=0.0781	
r-mTBI<br>♂ (n = 3, WT)<br>♂ (n = 4, Hemi)<br>♂ (n = 5, Hom)<br>♀ (n = 5, WT)<br>♀ (n = 5, Hemi)<br>♀ (n = 5, Hom)	Interaction	F(2, 20) = 0.3390; p=0.7165	WT Female vs. Male p=0.9091
	Sex	F(1, 20) = 0.0041; p=0.9497	Hemi Female vs. Male p=0.8751
	Genotype	F(2, 20) = 1.922; p=0.1725	Hom Female vs. Male p=0.8843
Cortex microglia<br>4 mths<br>IBA1	Interaction	F(2, 35) = 0.09953; p=0.9055	r-sham Hemi vs. Hom p=0.0976
	Injury	F(1, 35) = 0.5771; p=0.4526	r-mTBI WT vs. Hemi p=0.1157
	Genotype	F(2, 35) = 5.979; **p=0.0058**	r-mTBI Hemi vs. Hom p=0.1157
r-sham<br>♂ (n = 3, WT)<br>♂ (n = 4, Hemi)<br>♂ (n = 4, Hom)<br>♀ (n = 3, WT)<br>♀ (n = 6, Hemi)<br>♀ (n = 3, Hom)	Interaction	F(2, 17) = 0.06867; p=0.9339	WT Female vs. Male p=0.9828
	Sex	F(1, 17) = 0.0007; p=0.9790	Hemi Female vs. Male p=0.9825
	Genotype	F(2, 17) = 1.868; p=0.1848	Hom Female vs. Male p=0.9828
r-mTBI<br>♂ (n = 3, WT)<br>♂ (n = 4, Hemi)<br>♂ (n = 5, Hom)<br>♀ (n = 2, WT)<br>♀ (n = 5, Hemi)<br>♀ (n = 5, Hom)	Interaction	F(1, 15) = 0.3378; p=0.5698	Hemi Female vs. Male p=0.8318
	Sex	F(1, 15) = 0.03047; p=0.8638	Hom Female vs. Male p=0.8318
	Genotype	F(1, 15) = 2.229; p=0.1520	
CC astrocytes<br>6 wks<br>GFAP	Interaction	F(2, 42) = 1.418; p=0.2536	Hemi; r-sham vs. r-mTBI **p=0.0036**
	Injury	F(1, 42) = 14.32; **p=0.0005**	r-mTBI; WT vs. Hemi **p=0.0071**
	Genotype	F(2, 42) = 8.913; **p=0.0006**	r-mTBI; Hemi vs. Hom **p=0.0011**
r-sham<br>♂ (n = 4, WT)<br>♂ (n = 4, Hemi)	Interaction	F(2, 18) = 1.228; p=0.3161	WT Female vs. Male p=0.3536
	Sex	F(1, 18) = 2.956; p=0.1027	Hemi Female vs. Male p=0.2480
	Genotype	F(2, 18) = 5.082; **p=0.0178**	Hom Female vs. Male p=0.8131

♂ (n = 4, Hom)<br>♀ (n = 5, WT)<br>♀ (n = 6, Hemi)<br>♀ (n = 3, Hom)			
r-mTBI<br>♂ (n = 3, WT)<br>♂ (n = 4, Hemi)<br>♂ (n = 4, Hom)<br>♀ (n = 3, WT)<br>♀ (n = 5, Hemi)<br>♀ (n = 3, Hom)	Interaction	F(2, 18) = 0.255; p=0.7777	WT Female vs. Male p=0.2414
	Sex	F(1, 18) = 9.706; **p=0.0060**	Hemi Female vs. Male p=0.1040
	Genotype	F(2, 18) = 6.357; **p=0.0082**	Hom Female vs. Male p=0.2414
CC astrocytes<br>4 mths<br>GFAP	Interaction	F(2, 37) = 0.7277; p=0.4898	r-sham; WT vs. Hom p=0.9099
	Injury	F(1, 37) = 1.920; p=0.1742	r-mTBI; WT vs. Hemi p=0.9576
	Genotype	F(2, 37) =2.969; p=0.0637	r-mTBI; Hemi vs. Hom p=0.9576
r-sham<br>♂ (n = 3, WT)<br>♂ (n = 4, Hemi)<br>♂ (n = 4, Hom)<br>♀ (n = 3, WT)<br>♀ (n = 5, Hemi)<br>♀ (n = 3, Hom)	Interaction	F(2, 16) = 2.690; p=0.0984	WT Female vs. Male p=0.0544
	Sex	F(1, 16) = 7.157; **p=0.0166**	Hemi Female v.s Male p=0.0544
	Genotype	F(2, 16) = 4.312; **p=0.0318**	Hom Female vs. Male p=0.7252
r-mTBI<br>♂ (n = 3, WT)<br>♂ (n = 1, Hemi)<br>♂ (n = 4, Hom)<br>♀ (n = 2, WT)<br>♀ (n = 6, Hemi)<br>♀ (n = 5, Hom)		t-test	Hom Female vs. Male p=0.4713
Cortical astrocytes<br>6 wks<br>GFAP	Interaction	F(2, 42) = 0.8033; p=0.4546	r-sham Hemi vs. Hom p=0.5974
	Injury	F(1, 42) = 3.677; p=0.0620	r-mTBI WT vs. Hemi **p=0.0396**
	Genotype	F(2, 42) = 4.218; **p=0.0214**	r-mTBI Hemi vs. Hom **p=0.0396**
r-sham<br>♂ (n = 3, WT)<br>♂ (n = 4, Hemi)<br>♂ (n = 4, Hom)<br>♀ (n = 3, WT)<br>♀ (n = 5, Hemi)<br>♀ (n = 3, Hom)	Interaction	F(2, 18) = 1.187; p=0.3280	WT Female vs. Male p=0.5239
	Sex	F(1, 18) = 3.038; p=0.0984	Hemi Female vs. Male p=0.1664
	Genotype	F(2, 18) = 2.196; p=0.1402	Hom Female vs. Male p=0.9229
r-mTBI<br>♂ (n = 3, WT)<br>♂ (n = 3, Hemi)<br>♂ (n = 4, Hom)<br>♀ (n = 5, WT)<br>♀ (n = 4, Hemi)<br>♀ (n = 5, Hom)	Interaction	F(2, 18) = 0.4573; p=0.6402	WT Female vs. Male p=0.6668
	Sex	F(1, 18) = 2.374; p=0.1407	Hemi Female vs. Male p=0.3841
	Genotype	F(2, 18) = 2.331; p=0.1208	Hom Female vs. Male p=0.7929
Cortical astrocytes<br>4 mths<br>GFAP	Interaction	F(2, 39) = 0.4510; p=0.6403	r-sham WT vs. Hom p=0.5579
	Injury	F(1, 39) = 1.248; p=0.2707	r-sham Hemi vs. Hom **p=0.0198**
	Genotype	F(2, 39) = 5.231; **p=0.0097**	r-mTBI Hemi vs. Hom p=0.3218
r-sham<br>♂ (n = 3, WT)<br>♂ (n = 4, Hemi)	Interaction	F(2, 17) = 0.177; p=0.8386	WT Female vs. Male p=0.7745
	Sex	F(1, 17) = 2.480; p=0.1337	Hemi Female vs. Male p=0.3509
	Genotype	F(2, 17) = 2.807; p=0.0884	Hom Female vs. Male p=0.7745

♂ (n = 4, Hom)<br>♀ (n = 3, WT)<br>♀ (n = 6, Hemi)<br>♀ (n = 3, Hom)			
r-mTBI<br>♂ (n = 3, WT)<br>♂ (n = 3, Hemi)<br>♂ (n = 4, Hom)<br>♀ (n = 5, WT)<br>♀ (n = 4, Hemi)<br>♀ (n = 5, Hom)	Interaction	$F(2, 16) = 1.607$; $p=0.3674$	WT Female vs. Male $p=0.8808$
	Sex	$F(1, 16) = 2.583$; $p=0.1276$	Hemi Female vs. Male $p=0.2350$
	Genotype	$F(2, 16) = 0.2475$; $p=0.7837$	Hom Female vs. Male $p=0.8808$

Supplemental Information Table SI-8. Wes biochemical analysis for tau proteins in brain lysates. Statistical analysis for combined male and female mice to test a main effect of injury or time for each hTau.P301S genotype. Further analysis was conducted for sex as a biological variable with 3 or more mice, with text in gray for excluded comparisons that do not have at least 3 mice of both sexes.

**SI-8 r-mTBI**	**Main effect; F, p-value**		**Adjusted p-value**
HT7 (human Tau)	Interaction	F(4, 63) = 0.08570; p=0.9866	WT 4m vs. Hemi 6w **p=0.0041**
	Tau condition	F(4, 63) = 25.96; **p<0.0001**	WT 4m vs. Hemi 4m **p<0.0001**
	Injury	F(1, 63) = 1.285; p=0.2613	WT 4m vs. Hom 6w **p<0.0001**
			WT 4m vs. Hom 4m **p<0.0001**
			Hemi 6w vs. Hemi 4m p=0.1912
			Hemi 6w vs. Hom 6w **p<0.0001**
			Hemi 6w vs. Hom 4m **p=0.0004**
			Hemi 4m vs. Hom 6w **p=0.0002**
			Hemi 4m vs. Hom 4m **p=0.0230**
			Hom 6w vs. Hom 4m p=0.1912
Hemi<br>♂ (n=4, r-sham 6w)<br>♂ (n=4, r-sham 4m)<br>♂ (n=4, r-mTBI 6w)<br>♂ (n=5, r-mTBI 4m)<br>♀ (n=3, r-sham, 6w)<br>♀ (n=3, r-sham, 4m)<br>♀ (n=3, r-mTBI, 6w)<br>♀ (n=4, r-mTBI, 4m)	Interaction	F(3, 22) = 0.3516; p=0.3359	r-sham 6w F vs. M p=0.4511
	Sex	F(1, 22) = 5.627; **p=0.0269**	r-sham 4m F vs. M p=0.4511
	Injury	F(3, 22) = 0.9728; p=0.4233	r-mTBI 6w F vs. M p=0.4511
			r-mTBI 4m F vs. M p=0.7353
Hom<br>♂ (n=4, r-sham 6w)<br>♂ (n=4, r-sham 4m)<br>♂ (n=4, r-mTBI 6w)<br>♂ (n=5, r-mTBI 4m)<br>♀ (n=3, r-sham, 6w)<br>♀ (n=3, r-sham, 4m)<br>♀ (n=3, r-mTBI, 6w)<br>♀ (n=4, r-mTBI, 4m)	Interaction	F(3, 22) = 0.6756; p=0.5762	r-sham 6w F vs. M p=0.7349
	Sex	F(1, 22) = 5.805; **p=0.0248**	r-sham 4m F vs. M p=0.5887
	Injury	F(3, 22) = 1.372; p=0.2774	r-mTBI 6w F vs. M p=0.1059
			r-mTBI 4m F vs. M p=0.5440
Phospho-Tau at T231 (AT180)	Interaction	F(4, 58) = 0.4355; p=0.7824	WT 4m vs. Hemi 6w **p=0.0041**
	Tau condition	F(4, 58) = 22.26; p**<0.0001**	WT 4m vs. Hemi 4m **p<0.0001**
	Injury	F(1, 58) = 0.06223; p=0.8039	WT 4m vs. Hom 6w **p=0.0003**
			WT 4m vs. Hom 4m **p<0.0001**
			Hemi 6w vs. Hemi 4m p=0.4906
			Hemi 6w vs. Hom 6w p=0.5541
			Hemi 6w vs. Hom 4m **p<0.0001**
			Hemi 4m vs. Hom 6w p=0.8403
			Hemi 4m vs. Hom 4m **p=0.0001**
			Hom 6w vs. Hom 4m **p=0.0003**
Hemi<br>♂ (n=3, r-sham 6w)	Interaction	F(3, 21) = 0.1031; p=0.9574	r-sham 6w F vs. M p=0.9489
	Sex	F(1, 21) = 1.277; p=0.2712	r-sham 4m F vs. M p=0.7932

♂ (n=4, r-sham 4m)<br>♂ (n=3, r-mTBI 6w)<br>♂ (n=5, r-mTBI 4m)<br>♀ (n=3, r-sham, 6w)<br>♀ (n=3, r-sham, 4m)<br>♀ (n=3, r-mTBI, 6w)<br>♀ (n=4, r-mTBI, 4m)	Injury	F(3, 21) = 0.6715; p=0.5791	r-mTBI 6w F vs. M p=0.9489
			r-mTBI 4m F vs. M p=0.9489
Hom<br>♂ (n=3, r-sham 6w)<br>♂ (n=3, r-sham 4m)<br>♂ (n=4, r-mTBI 6w)<br>♂ (n=4, r-mTBI 4m)<br>♀ (n=2, r-sham, 6w)<br>♀ (n=4, r-sham, 4m)<br>♀ (n=2, r-mTBI, 6w)<br>♀ (n=4, r-mTBI, 4m)	Interaction	F(1, 11) = 0.002576; p=0.9604	r-sham 4m F vs. M p=0.5040
	Sex	F(1, 11) = 2.379; p=0.1513	r-mTBI 4m F vs. M p=0.5040
	Injury	F(1, 11) = 0.7115; p=0.4169	
Phospho-Tau at S404	Interaction	F(4, 58) = 0.2640; p=0.8999	WT 4m vs. Hemi 6w **p<0.0001**
	Tau condition	F(4, 58) = 58.88; **p<0.0001**	WT 4m vs. Hemi 4m **p<0.0001**
	Injury	F(1, 58) = 0.2197; p=0.6411	WT 4m vs. Hom 6w **p<0.0001**
			WT 4m vs. Hom 4m **p<0.0001**
			Hemi 6w vs. Hemi 4m p=0.3358
			Hemi 6w vs. Hom 6w **p<0.0001**
			Hemi 6w vs. Hom 4m p=0.6285
			Hemi 4m vs. Hom 6w **p<0.0001**
			Hemi 4m vs. Hom 4m p=0.6285
			Hom 6w vs. Hom 4m **p<0.0001**
WT<br>♂ (n=4, r-sham 4m)<br>♂ (n=3, r-mTBI 4m)<br>♀ (n=3, r-sham, 4m)<br>♀ (n=3, r-mTBI, 4m)	Interaction	F(1, 9) = 2.055; p=0.1856	r-sham 4m F vs. M p=0.2799
	Sex	F(1, 9) = 9.025; **p=0.0149**	r-mTBI 4m F vs. M **p=0.0279**
	Injury	F(1, 9) = 1.274; p=0.2883	
Hemi<br>♂ (n=4, r-sham 6w)<br>♂ (n=3, r-sham 4m)<br>♂ (n=4, r-mTBI 6w)<br>♂ (n=3, r-mTBI 4m)<br>♀ (n=3, r-sham, 6w)<br>♀ (n=3, r-sham, 4m)<br>♀ (n=3, r-mTBI, 6w)<br>♀ (n=3, r-mTBI, 4m)	Interaction	F(3, 18) = 0.443; p=0.7249	r-sham 6w F vs. M p=0.9991
	Sex	F(1, 18) = 1.411; p=0.2503	r-sham 4m F vs. M p=0.9991
	Injury	F(3, 18) = 1.442; p=0.2637	r-mTBI 6w F vs. M p=0.6848
			r-mTBI 4m F vs. M p=0.6848
Hom<br>♂ (n=3, r-sham 6w)<br>♂ (n=3, r-sham 4m)<br>♂ (n=3, r-mTBI 6w)<br>♂ (n=4, r-mTBI 4m)<br>♀ (n=4, r-sham, 6w)<br>♀ (n=4, r-sham, 4m)<br>♀ (n=4, r-mTBI, 6w)<br>♀ (n=4, r-mTBI, 4m)	Interaction	F(3, 21) = 0.4480; p=0.7213	r-sham 6w F vs. M p=0.4466
	Sex	F(1, 21) = 9.240; **p=0.0062**	r-sham 4m F vs. M p=0.3991
	Injury	F(3, 21) = 20.67; **p<0.0001**	r-mTBI 6w F vs. M p=0.1061
			r-mTBI 4m F vs. M p=0.2786
Phospho-Tau at T205	Interaction	F(4, 63) = 0.4411; p=0.7784	WT 4m vs. Hemi 6w p=0.4147

	Tau condition	F(4, 63) = 10.43; **p<0.0001**	WT 4m vs. Hemi 4m **p=0.0006**
	Injury	F(1, 63) = 0.3526; p=0.5548	WT 4m vs. Hom 6w **p<0.0001**
			WT 4m vs. Hom 4m **p<0.0001**
			Hemi 6w vs. Hemi 4m **p=0.0462**
			Hemi 6w vs. Hom 6w **p=0.0089**
			Hemi 6w vs. Hom 4m **p=0.0022**
			Hemi 4m vs. Hom 6w p=0.6717
			Hemi 4m vs. Hom 4m p=0.5056
			Hom 6w vs. Hom 4m p=0.6717
WT<br>♂ (n=4, r-sham 4m)<br>♂ (n=3, r-mTBI 4m)<br>♀ (n=3, r-sham, 4m)<br>♀ (n=3, r-mTBI, 4m)	Interaction	F(1, 9) = 1.630; p=0.2337	r-sham 4m F vs. M p=0.9890
	Sex	F(1, 9) = 1.680; p=0.2272	r-mTBI 4m F vs. M p=0.2115
	Injury	F(1, 9) = 1.735; p=0.2203	
Hemi<br>♂ (n=4, r-sham 6w)<br>♂ (n=4, r-sham 4m)<br>♂ (n=4, r-mTBI 6w)<br>♂ (n=5, r-mTBI 4m)<br>♀ (n=3, r-sham, 6w)<br>♀ (n=3, r-sham, 4m)<br>♀ (n=3, r-mTBI, 6w)<br>♀ (n=4, r-mTBI, 4m)	Interaction	F(3, 23) = 0.08676; p=0.9666	r-sham 6w F vs. M p=0.9589
	Sex	F(1, 23) = 0.1610; p=0.6919	r-sham 4m F vs. M p=0.9988
	Injury	F(3, 23) = 1.695; p=0.1958	r-mTBI 6w F vs. M p=0.9988
			r-mTBI 4m F vs. M p=0.9954
Hom<br>♂ (n=4, r-sham 6w)<br>♂ (n=3, r-sham 4m)<br>♂ (n=3, r-mTBI 6w)<br>♂ (n=4, r-mTBI 4m)<br>♀ (n=3, r-sham, 6w)<br>♀ (n=4, r-sham, 4m)<br>♀ (n=4, r-mTBI, 6w)<br>♀ (n=4, r-mTBI, 4m)	Interaction	F(3, 21) = 1.358; p=0.5762	r-sham 6w F vs. M p=0.9494
	Sex	F(1, 21) = 0.2505; p=0.6219	r-sham 4m F vs. M p=0.5891
	Injury	F(3, 21) = 0.1321; p=0.9399	r-mTBI 6w F vs. M p=0.3626
			r-mTBI 4m F vs. M p=0.9494
**SI-8 s-TBI**	**Main effect; F, p-value**		**Adjusted p-value**
HT7(human Tau)	Interaction	F(4, 51) = 0.5768; p=0.6807	WT 4m vs. Hemi 6w **p=0.0097**
	Tau condition	F(4, 51) = 15.33; **p<0.0001**	WT 4m vs. Hemi 4m **p=0.0445**
	Injury	F(1, 51) = 0.06845; p=0.7947	WT 4m vs. Hom 6w **p<0.0001**
			WT 4m vs. Hom 4m **p=0.0048**
			Hemi 6w vs. Hemi 4m p=0.7383
			Hemi 6w vs. Hom 6w **p=0.0004**
			Hemi 6w vs. Hom 4m p=0.7649
			Hemi 4m vs. Hom 6w **p<0.0001**
			Hemi 4m vs. Hom 4m p=0.6857
			Hom 6w vs. Hom 4m **p=0.0010**
Hemi	Interaction	F(3, 17) = 1.211; p=0.3359	s-sham 6w F vs. M p=0.8237

♂ (n=3, s-sham 6w)<br>♂ (n=3, s-sham 4m)<br>♂ (n=3, s-TBI 6w)<br>♂ (n=3, s-TBI 4m)<br>♀ (n=3, s-sham, 6w)<br>♀ (n=3, s-sham, 4m)<br>♀ (n=3, s-TBI, 6w)<br>♀ (n=4, s-TBI, 4m)	Sex	F(1, 17) = 0.3484; p=0.5628	s-sham 4m F vs. M p=0.7011
	Injury	F(3, 17) = 0.3996; p=0.7550	s-TBI 6w F vs. M p=0.4077
			s-TBI 4m F vs. M p=0.9778
Hom<br>♂ (n=3, s-sham 6w)<br>♂ (n=3, s-sham 4m)<br>♂ (n=3, s-TBI 6w)<br>♂ (n=3, s-TBI 4m)<br>♀ (n=3, s-sham, 6w)<br>♀ (n=3, s-sham, 4m)<br>♀ (n=3, s-TBI, 6w)<br>♀ (n=3, s-TBI, 4m)	Interaction	F(3, 16) = 0.0038; p=0.3359	s-sham 6w F vs. M p=0.9925
	Sex	F(1, 16) = 0.4355; p=0.5187	s-sham 4m F vs. M p=0.9925
	Injury	F(3, 16) = 2.667; p=0.0829	s-TBI 6w F vs. M p=0.9925
			s-TBI 4m F vs. M p=0.9925
AT180 (phospho-Tau at T231)	Interaction	F(4, 51) = 0.1707; p=0.9524	WT 4m vs. Hemi 6w p=0.1306
	Tau condition	F(4, 51) = 38.80; **p<0.0001**	WT 4m vs. Hemi 4m p=0.1519
	Injury	F(1, 51) = 0.8726; p=0.3546	WT 4m vs. Hom 6w **p<0.0001**
			WT 4m vs. Hom 4m **p<0.0001**
			Hemi 6w vs. Hemi 4m p=0.8613
			Hemi 6w vs. Hom 6w **p<0.0001**
			Hemi 6w vs. Hom 4m **p<0.0001**
			Hemi 4m vs. Hom 6w **p<0.0001**
			Hemi 4m vs. Hom 4m **p<0.0001**
			Hom 6w vs. Hom 4m p=0.8613
Hemi<br>♂ (n=3, s-sham 6w)<br>♂ (n=3, s-sham 4m)<br>♂ (n=3, s-TBI 6w)<br>♂ (n=3, s-TBI 4m)<br>♀ (n=3, s-sham, 6w)<br>♀ (n=3, s-sham, 4m)<br>♀ (n=3, s-TBI, 6w)<br>♀ (n=4, s-TBI, 4m)	Interaction	F(3, 17) = 0.3586; p=0.7836	s-sham 6w F vs. M p=0.9334
	Sex	F(1, 17) = 0.4529; p=0.5100	s-sham 4m F vs. M p=0.9697
	Injury	F(3, 17) = 1.193; p=0.3422	s-TBI 6w F vs. M p=0.7632
			s-TBI 4m F vs. M p=0.9697
Hom<br>♂ (n=3, s-sham 6w)<br>♂ (n=3, s-sham 4m)<br>♂ (n=3, s-TBI 6w)<br>♂ (n=3, s-TBI 4m)<br>♀ (n=3, s-sham, 6w)<br>♀ (n=3, s-sham, 4m)<br>♀ (n=3, s-TBI, 6w)<br>♀ (n=3, s-TBI, 4m)	Interaction	F(3, 16) = 0.4065; p=0.7504	s-sham 6w F vs. M p=0.8013
	Sex	F(1, 16) = 3.822; p=0.0683	s-sham 4m F vs. M p=0.8013
	Injury	F(3, 16) = 0.1288; p=0.9416	s-TBI 6w F vs. M p=0.8013
			s-TBI 4m F vs. M p=0.2755
Phospho-Tau at S404	Interaction	F(4, 51) = 0.09388; p=0.9840	WT 4m vs. Hemi 6w **p<0.0001**
	Tau condition	F(4, 51) = 32.42; **p<0.0001**	WT 4m vs. Hemi 4m **p=0.0021**
	Injury	F(1, 51) = 0.0086; p=0.9264	WT 4m vs. Hom 6w **p<0.0001**

			WT 4m vs. Hom 4m **p=0.0007**
			Hemi 6w vs. Hemi 4m p=0.2111
			Hemi 6w vs. Hom 6w **p<0.0001**
			Hemi 6w vs. Hom 4m p=0.3687
			Hemi 4m vs. Hom 6w **p<0.0001**
			Hemi 4m vs. Hom 4m p=0.6156
			Hom 6w vs. Hom 4m **p<0.0001**
WT<br>♂ (n=3, s-sham 4m)<br>♂ (n=3, s-TBI 4m)<br>♀ (n=3, s-sham, 4m)<br>♀ (n=3, s-TBI, 4m)	Interaction	F(1, 8) = 0.05722; p=0.8170	s-sham 4m F vs. M p=0.9040
	Sex	F(1, 8) = 0.1725; p=0.6888	s-TBI 4m F vs. M p=0.8815
	Injury	F(1, 8) = 0.3089; p=0.5935	
Hemi<br>♂ (n=3, s-sham 6w)<br>♂ (n=3, s-sham 4m)<br>♂ (n=3, s-TBI 6w)<br>♂ (n=3, s-TBI 4m)<br>♀ (n=3, s-sham, 6w)<br>♀ (n=3, s-sham, 4m)<br>♀ (n=3, s-TBI, 6w)<br>♀ (n=4, s-TBI, 4m)	Interaction	F(3, 17) = 0.5750; p=0.6392	s-sham 6w F vs. M p=0.2838
	Sex	F(1, 17) = 2.887; p=0.1075	s-sham 4m F vs. M p=0.8600
	Injury	F(3, 17) = 3.278; **p=0.0466**	s-TBI 6w F vs. M p=0.8600
			s-TBI 4m F vs. M p=0.9245
Hom<br>♂ (n=3, s-sham 6w)<br>♂ (n=3, s-sham 4m)<br>♂ (n=3, s-TBI 6w)<br>♂ (n=3, s-TBI 4m)<br>♀ (n=3, s-sham, 6w)<br>♀ (n=3, s-sham, 4m)<br>♀ (n=3, s-TBI, 6w)<br>♀ (n=3, s-TBI, 4m)	Interaction	F(3, 16) = 0.6694; p=0.5831	s-sham 6w F vs. M p=0.8270
	Sex	F(1, 16) = 0.1762; p=0.6802	s-sham 4m F vs. M p=0.8270
	Injury	F(3, 16) = 6.790; **p=0.0037**	s-TBI 6w F vs. M p=0.8270
			s-TBI 4m F vs. M p=0.8270
Phospho-Tau at T205	Interaction	F(4, 51) = 0.4398; p=0.7792	WT 4m vs. Hemi 6w **p=0.0001**
	Tau condition	F(4, 51) = 33.30; **p<0.0001**	WT 4m vs. Hemi 4m **p=0.0012**
	Injury	F(1, 51) = 0.05963; p=0.8081	WT 4m vs. Hom 6w **p<0.0001**
			WT 4m vs. Hom 4m **p=0.0012**
			Hemi 6w vs. Hemi 4m p=0.7537
			Hemi 6w vs. Hom 6w **p<0.0001**
			Hemi 6w vs. Hom 4m p=0.7537
			Hemi 4m vs. Hom 6w **p<0.0001**
			Hemi 4m vs. Hom 4m p=0.8794
			Hom 6w vs. Hom 4m **p<0.0001**
WT<br>♂ (n=3, s-sham 4m)	Interaction	F(1, 8) = 0.4272; p=0.5317	s-sham 4m F vs. M p=0.7370
	Sex	F(1, 8) = 0.1418; p=0.7163	s-TBI 4m F vs. M p=0.8496

♂ (n=3, s-TBI 4m)<br>♀ (n=3, s-sham, 4m)<br>♀ (n=3, s-TBI, 4m)	Injury	F(1, 8) = 0.9954; p=0.3476	
Hemi<br>♂ (n=3, s-sham 6w)<br>♂ (n=3, s-sham 4m)<br>♂ (n=3, s-TBI 6w)<br>♂ (n=3, s-TBI 4m)<br>♀ (n=3, s-sham, 6w)<br>♀ (n=3, s-sham, 4m)<br>♀ (n=3, s-TBI, 6w)<br>♀ (n=4, s-TBI, 4m)	Interaction	F(3, 17) = 1.841; p=0.1779	s-sham 6w F vs. M p=0.0606
	Sex	F(1, 17) = 2.355; p=0.1433	s-sham 4m F vs. M p=0.9593
	Injury	F(3, 17) = 9.579; **p=0.0006**	s-TBI 6w F vs. M p=0.9593
			s-TBI 4m F vs. M p=0.9593
Hom<br>♂ (n=3, s-sham 6w)<br>♂ (n=3, s-sham 4m)<br>♂ (n=3, s-TBI 6w)<br>♂ (n=3, s-TBI 4m)<br>♀ (n=3, s-sham, 6w)<br>♀ (n=3, s-sham, 4m)<br>♀ (n=3, s-TBI, 6w)<br>♀ (n=3, s-TBI, 4m)	Interaction	F(3, 16) = 2.318; p=0.1143	s-sham 6w F vs. M **p=0.0493**
	Sex	F(1, 16) = 8.387; **p=0.0105**	s-sham 4m F vs. M p=0.7155
	Injury	F(3, 16) = 14.21; **p<0.0001**	s-TBI 6w F vs. M p=0.0571
			s-TBI 4m F vs. M p=0.7214

Supplemental Information Table SI-9. Simoa™ analysis of serum tau and neurofilament light protein. Statistical analysis for combined male and female mice to test a main effect of injury or hTau.P301S genotype for mice at each post-injury time point (1 day, 6 weeks, or 4 months). Further analysis was conducted for sex as a biological variable with 3 or more mice, with text in gray for excluded comparisons that do not have at least 3 mice of both sexes.

**SI-9 r-mTBI**	**Main effect; F, p-value**		**Adjusted p-value**
Total human Tau<br>1 dy	Interaction	F(2, 42) = 1.515; p=0.2316	r-sham Wt vs. Hemi **p=0.0127**
	Injury	F(1, 42) = 0.02539; p=0.8742	r-sham Wt vs. Hom **p=0.0127**
	Genotype	F(2, 42) = 14.38; **p<0.0001**	r-sham Hemi vs. Hom p=0.7041
			r-mTBI Wt vs. Hemi p=0.0500
			r-mTBI Wt vs. Hom **p=0.0001**
			r-mTBI Hemi vs. Hom **p=0.0137**
r-sham<br>♂ (n = 4, WT)<br>♂ (n = 7, Hemi)<br>♂ (n = 4, Hom)<br>♀ (n = 4, WT)<br>♀ (n = 6, Hemi)<br>♀ (n = 3, Hom)	Interaction	F(2, 18) = 1.316; p=0.2852	WT Female vs. Male p>0.9999
	Sex	F(1, 18) = 5.474; **p=0.0310**	Hemi Female vs. Male p=0.1623
	Genotype	F(2, 18) = 5.520; **p=0.0135**	Hom Female vs. Male p=0.0621
r-mTBI<br>♂ (n = 4, WT)<br>♂ (n = 3, Hemi)<br>♂ (n = 4, Hom)<br>♀ (n = 4, WT)<br>♀ (n = 7, Hemi)<br>♀ (n = 3, Hom)	Interaction	F(2, 19) = 1.962; p=0.1680	WT Female vs. Male p>0.9999
	Sex	F(1, 19) = 2.790; p=0.1113	Hemi Female vs. Male p=0.9525
	Genotype	F(2, 19) = 28.70; **p<0.0001**	Hom Female vs. Male p=0.0668
Neurofilament light<br>1 dy	Interaction	F(2, 46) = 0.2289; p=0.7963	r-sham Wt vs. Hemi p=0.6906
	Genotype	F(1, 46) = 15.84; **p=0.0002**	r-sham Wt vs. Hom **p=0.0205**
	Injury	F(2, 46) = 10.46; **p=0.0002**	r-sham Hemi vs. Hom **p=0.0057**
			r-mTBI Wt vs. Hemi p=0.2460
			r-mTBI Wt vs. Hom p=0.1678
			r-mTBI Hemi vs. Hom **p=0.0098**
			Wt r-sham vs. r-mTBI **p=0.0269**
			Hemi r-sham vs. r-mTBI **p=0.0354**
			Hom r-sham vs. r-mTBI p=0.0794
r-sham<br>♂ (n = 4, WT)<br>♂ (n = 8, Hemi)<br>♂ (n = 4, Hom)<br>♀ (n = 4, WT)<br>♀ (n = 5, Hemi)<br>♀ (n = 3, Hom)	Interaction	F(2, 21) = 0.7093; p=0.5034	WT Female vs. Male p=0.9835
	Sex	F(1, 21) = 0.7501; p=0.3962	Hemi Female vs. Male p=0.3333
	Genotype	F(2, 21) = 13.74; **p=0.0002**	Hom Female vs. Male p=0.9835
r-mTBI<br>♂ (n = 4, WT)<br>♂ (n = 6, Hemi)<br>♂ (n = 4, Hom)<br>♀ (n = 4, WT)<br>♀ (n = 5, Hemi)	Interaction	F(2, 19) = 2.571; p=0.1027	WT Female vs. Male p=0.0840
	Sex	F(1, 19) = 1.296; p=0.2691	Hemi Female vs. Male p=0.8547
	Genotype	F(2, 19) = 3.412; p=0.0542	Hom Female vs. Male p=0.9071

♀ (n = 3, Hom)			
Total human Tau<br>6 wks	Interaction	F(2, 38) = 2.131; p=0.1327	WT 6w vs. Hemi 6w **p=0.0005**
	Genotype	F(2, 38) = 9.284; **p=0.0005**	WT 6w vs. Hom 6w **p=0.0107**
	Injury	F(1, 38) = 3.804; p=0.0585	Hemi 6w vs. Hom 6w p=0.2735
r-sham<br>♂ (n = 5, WT)<br>♂ (n = 5, Hemi)<br>♂ (n = 4, Hom)<br>♀ (n = 5, WT)<br>♀ (n = 6, Hemi)<br>♀ (n = 3, Hom)	Interaction	F(2, 20) = 1.029; p=0.3754	WT Female vs. Male p=0.9784
	Sex	F(1, 20) = 2.626; p=0.1208	Hemi Female vs. Male p=0.2480
	Genotype	F(2, 20) = 8.319; **p=0.0023**	Hom Female vs. Male p=0.6682
r-mTBI<br>♂ (n = 3, WT)<br>♂ (n = 4, Hemi)<br>♂ (n = 4, Hom)<br>♀ (n = 4, WT)<br>♀ (n = 6, Hemi)<br>♀ (n = 6, Hom)	Interaction	F(2, 13) = 0.1475; p=0.8643	WT Female vs. Male p=0.9981
	Sex	F(1, 13) = 0.3088; p=0.5879	Hemi Female vs. Male p=0.8805
	Genotype	F(2, 13) = 0.2046; p=0.8176	Hom Female vs. Male p=0.9758
Neurofilament light<br>6 wks	Interaction	F(2, 53) = 0.3639; p=0.6967	WT 6w vs. Hemi 6w p=0.4764
	Genotype	F(2, 53) = 34.83; **p<0.0001**	WT 6w vs. Hom 6w **p<0.0001**
	Injury	F(1, 53) = 0.6061; p=0.4397	Hemi 6w vs. Hom 6w **p<0.0001**
r-sham<br>♂ (n = 5, WT)<br>♂ (n = 4, Hemi)<br>♂ (n = 7, Hom)<br>♀ (n = 5, WT)<br>♀ (n = 7, Hemi)<br>♀ (n = 5, Hom)	Interaction	F(2, 27) = 1.273; p=0.2963	WT Female vs. Male p=0.4809
	Sex	F(1, 27) = 2.326; p=0.1389	Hemi Female vs. Male p=0.7373
	Genotype	F(2, 27) = 37.8; **p<0.0001**	Hom Female vs. Male p=0.1749
r-mTBI<br>♂ (n = 4, WT)<br>♂ (n = 3, Hemi)<br>♂ (n = 4, Hom)<br>♀ (n = 3, WT)<br>♀ (n = 7, Hemi)<br>♀ (n = 6, Hom)	Interaction	F(2, 20) = 1.342; p=0.2838	WT Female vs. Male p=0.2200
	Sex	F(1, 20) = 1.451; p=0.2424	Hemi Female vs. Male p=0.8289
	Genotype	F(2, 20) = 7.321; **p=0.0041**	Hom Female vs. Male p=0.8289
Total human Tau<br>4 mths	Interaction	F(2, 87) = 0.6698; p=0.5144	WT 4m vs. Hemi 4m p=0.6718
	Genotype	F(2, 87) = 15.64; **p<0.0001**	WT 4m vs. Hom 4m **p<0.0001**
	Injury	F(1, 87) = 0.5457; p=0.4621	Hemi 4m vs. Hom 4m **p<0.0001**
r-sham<br>♂ (n = 8, WT)<br>♂ (n = 9, Hemi)<br>♂ (n = 7, Hom)<br>♀ (n = 7, WT)<br>♀ (n = 10, Hemi)<br>♀ (n = 7, Hom)	Interaction	F(2, 40) = 0.0064; p=0.9936	WT Female vs. Male p>0.9999
	Sex	F(1, 40) = 0.02037; p=0.8872	Hemi Female vs. Male p=0.9976
	Genotype	F(2, 40) = 6.288; **p=0.0042**	Hom Female vs. Male p=0.9976
r-mTBI<br>♂ (n = 9, WT)<br>♂ (n = 10, Hemi)	Interaction	F(2, 41) = 0.2406; p=0.7873	WT Female vs. Male p=0.9648
	Sex	F(1, 41) = 0.2431; p=0.6246	Hemi Female vs. Male p=0.9648
	Genotype	F(2, 41) = 11.68; **p<0.0001**	Hom Female vs. Male p=0.7915

♂ (n = 9, Hom)<br>♀ (n = 8, WT)<br>♀ (n = 10, Hemi)<br>♀ (n = 9, Hom)					
Neurofilament light 4 mths	Interaction	F(2, 98) = 0.3701;	p=0.6916	WT 4m vs. Hemi 4m	p=0.9193
	Genotype	F(2, 98) = 229.1;	**p<0.0001**	WT 4m vs. Hom 4m	**p<0.0001**
	Injury	F(1, 98) = 0.2110;	p=0.6470	Hemi 4m vs. Hom 4m	**p<0.0001**
r-sham<br>♂ (n = 7, WT)<br>♂ (n = 10, Hemi)<br>♂ (n = 7, Hom)<br>♀ (n = 8, WT)<br>♀ (n = 10, Hemi)<br>♀ (n = 7, Hom)	Interaction	F(2, 43) = 0.00570;	p=0.9446	WT Female vs. Male	p=0.9923
	Sex	F(1, 43) = 0.00036;	p=0.9849	Hemi Female vs. Male	p=0.9923
	Genotype	F(2, 43) = 85.70;	**p<0.0001**	Hom Female vs. Male	p=0.9923
r-mTBI<br>♂ (n = 9, WT)<br>♂ (n = 10, Hemi)<br>♂ (n = 9, Hom)<br>♀ (n = 8, WT)<br>♀ (n = 10, Hemi)<br>♀ (n = 9, Hom)	Interaction	F(2, 49) = 0.4734;	p=0.6257	WT Female vs. Male	p=0.9926
	Sex	F(1, 49) = 0.5933;	p=0.4448	Hemi Female vs. Male	p=0.9926
	Genotype	F(2, 49) = 139.6;	**p<0.0001**	Hom Female vs. Male	p=0.5314
**s-TBI**	**Main effect; F, p-value**			**Adjusted p-value**	
Total human Tau 1 dy	Interaction	F(2, 35) = 2.492;	p=0.0973	s-sham Wt vs. Hemi	p=0.9086
	Injury	F(1, 35) = 10.49;	**p=0.0026**	s-sham Wt vs. Hom	p=0.7065
	Genotype	F(2, 35) = 6.752;	**p=0.0033**	s-sham Hemi vs. Hom	p=0.7065
				s-TBI Wt vs. Hemi	p=0.0763
				s-TBI Wt vs. Hom	**p=0.0008**
				s-TBI Hemi vs. Hom	p=0.0693
				Wt s-sham vs. s-TBI	p=0.8620
				Hemi s-sham vs. s-TBI	p=0.0910
				Hom s-sham vs. s-TBI	**p=0.0017**
s-sham<br>♂ (n = 4, WT)<br>♂ (n = 4, Hemi)<br>♂ (n = 7, Hom)<br>♀ (n = 2, WT)<br>♀ (n = 4, Hemi)<br>♀ (n = 3, Hom)	Interaction	F(1, 11) = 1.798;	p=0.2070	Hemi Female vs. Male	p=0.9769
	Sex	F(1, 11) = 1.908;	p=0.1946	Hom Female vs. Male	p=0.1705
	Genotype	F(1, 11) = 45.61;	p<0.0001		
s-TBI<br>♂ (n = 4, WT)<br>♂ (n = 3, Hemi)<br>♂ (n = 6, Hom)<br>♀ (n = 4, WT)<br>♀ (n = 4, Hemi)<br>♀ (n = 4, Hom)	Interaction	F(2, 14) = 1.365;	p=0.2874	WT Female vs. Male	p=0.8741
	Sex	F(1, 14) = 0.1005;	p=0.7559	Hemi Female vs. Male	p=0.5313
	Genotype	F(2, 14) = 3.238;	p=0.0698	Hom Female vs. Male	p=0.5313
Neurofilament light 1 dy	Interaction	F(2, 38) = 2.202;	p=0.1244	Wt s-sham vs. s-TBI	**p=0.0003**
	Injury	F(2, 38) = 25.86;	**p<0.0001**	Hemi s-sham vs. s-TBI	**p=0.0411**
	Genotype	F(1, 38) = 1.416;	p=0.2551	Hom s-sham vs. s-TBI	p=0.0629

s-sham<br>♂ (n = 5, WT)<br>♂ (n = 4, Hemi)<br>♂ (n = 7, Hom)<br>♀ (n = 2, WT)<br>♀ (n = 4, Hemi)<br>♀ (n = 3, Hom)	Interaction	F(1, 12) = 1.525; p=0.2404	Hemi Female vs. Male p=0.5066
	Sex	F(1, 12) = 0.1317; p=0.7229	Hom Female vs. Male p=0.5330
	Genotype	F(1, 12) = 2.305; p=0.1792	
s-TBI<br>♂ (n = 4, WT)<br>♂ (n = 3, Hemi)<br>♂ (n = 6, Hom)<br>♀ (n = 4, WT)<br>♀ (n = 4, Hemi)<br>♀ (n = 4, Hom)	Interaction	F(2, 16) = 0.1714; p=0.8440	WT Female vs. Male p=0.8381
	Sex	F(1, 16) = 1.209; p=0.2879	Hemi Female vs. Male p=0.6737
	Genotype	F(2, 16) = 1.564; p=0.2397	Hom Female vs. Male p=0.8381
Total human Tau<br>6 wks	Interaction	F(2, 60) = 0.1863; p=0.8305	WT 6w vs. Hemi 6w p=0.8658
	Genotype	F(2, 60) = 2.369; p=0.1023	WT 6w vs. Hom 6w p=0.1854
	Injury	F(1, 60) = 0.1186; p=0.7318	Hemi 6w vs. Hom 6w p=0.1854
s-sham<br>♂ (n = 3, WT)<br>♂ (n = 9, Hemi)<br>♂ (n = 8, Hom)<br>♀ (n = 4, WT)<br>♀ (n = 7, Hemi)<br>♀ (n = 6, Hom)	Interaction	F(2, 25) = 0.6315; p=0.5401	WT Female vs. Male p>0.9999
	Sex	F(1, 25) = 0.5409; p=0.4689	Hemi Female vs. Male p>0.9999
	Genotype	F(2, 25) = 0.9931; p=0.3846	Hom Female vs. Male p=0.4135
s-TBI<br>♂ (n = 7, WT)<br>♂ (n = 10, Hemi)<br>♂ (n = 8, Hom)<br>♀ (n = 4, WT)<br>♀ (n = 6, Hemi)<br>♀ (n = 6, Hom)	Interaction	F(2, 28) = 1.088; p=0.3570	WT Female vs. Male p=0.9952
	Sex	F(1, 28) = 1.357; p=0.2538	Hemi Female vs. Male p=0.9891
	Genotype	F(2, 28) = 1.447; p=0.2524	Hom Female vs. Male p=0.2144
Neurofilament light<br>6 wks	Interaction	F(2, 70) = 0.2562; p=0.7747	WT 6w vs. Hemi 6w p=0.6839
	Genotype	F(2, 70) = 71.33; **p<0.0001**	WT 6w vs. Hom 6w **p<0.0001**
	Injury	F(1, 70) = 6.024; **p=0.0166**	Hemi 6w vs. Hom 6w **p<0.0001**
s-sham<br>♂ (n = 3, WT)<br>♂ (n = 9, Hemi)<br>♂ (n = 8, Hom)<br>♀ (n = 4, WT)<br>♀ (n = 7, Hemi)<br>♀ (n = 6, Hom)	Interaction	F(2, 31) = 1.194; p=0.3167	WT Female vs. Male p=0.4942
	Sex	F(1, 31) = 5.297; **p=0.0282**	Hemi Female vs. Male p=0.6366
	Genotype	F(2, 31) = 25.65; **p<0.0001**	Hom Female vs. Male **p=0.0463**
s-TBI<br>♂ (n = 8, WT)<br>♂ (n = 10, Hemi)<br>♂ (n = 8, Hom)<br>♀ (n = 6, WT)<br>♀ (n = 6, Hemi)<br>♀ (n = 3, Hom)	Interaction	F(2, 33) = 1.399; p=0.2612	WT Female vs. Male p=0.2697
	Sex	F(1, 33) = 4.440; p=0.0428	Hemi Female vs. Male p=0.9648
	Genotype	F(2, 33) = 52.5; **p<0.0001**	Hom Female vs. Male p=0.1560
Total human Tau<br>4 mths	Interaction	F(2, 53) = 1.565; p=0.2186	WT 4m vs. Hemi 4m p=0.1020
	Genotype	F(2, 53) = 5.115; **p=0.0093**	WT 4m vs. Hom 4m **p=0.0070**

	Injury	F(1, 53) = 0.0139; p=0.9066	Hemi 4m vs. Hom 4m p=0.2771
s-sham<br>♂ (n = 4, WT)<br>♂ (n = 7, Hemi)<br>♂ (n = 8, Hom)<br>♀ (n = 6, WT)<br>♀ (n = 5, Hemi)<br>♀ (n = 7, Hom)	Interaction<br>Sex<br>Genotype	F(2, 23) = 2.059; p=0.1504<br>F(1, 23) = 4.191; p=0.0522<br>F(2, 23) = 2.702; p=0.0833	WT Female vs. Male p=0.9181<br>Hemi Female vs. Male p=0.0470<br>Hom Female vs. Male p=0.9181
s-TBI<br>♂ (n = 4, WT)<br>♂ (n = 8, Hemi)<br>♂ (n = 7, Hom)<br>♀ (n = 5, WT)<br>♀ (n = 7, Hemi)<br>♀ (n = 7, Hom)	Interaction<br>Sex<br>Genotype	F(2, 24) = 0.04094; p=0.9600<br>F(1, 24) = 0.2324; p=0.6341<br>F(2, 24) = 3.392; p=0.0504	WT Female vs. Male p=0.9856<br>Hemi Female vs. Male p=0.9403<br>Hom Female vs. Male p=0.9403
Neurofilament light<br>4 mths	Interaction<br>Genotype<br>Injury	F(2, 66) = 0.2626; **p=0.7698**<br>F(2, 66) = 73.29; **p<0.0001**<br>F(1, 66) = 0.2656; **p=0.6080**	WT 4m vs. Hemi 4m p=0.9579<br>WT 4m vs. Hom 4m **p<0.0001**<br>Hemi 4m vs. Hom 4m **p<0.0001**
s-sham<br>♂ (n = 3, WT)<br>♂ (n = 7, Hemi)<br>♂ (n = 8, Hom)<br>♀ (n = 6, WT)<br>♀ (n = 5, Hemi)<br>♀ (n = 7, Hom)	Interaction<br>Sex<br>Genotype	F(2, 30) = 0.2990; p=0.7437<br>F(1, 30) = 0.1731; p=0.6804<br>F(2, 30) = 51.45; **p<0.0001**	WT Female vs. Male p=0.9970<br>Hemi Female vs. Male p=0.9970<br>Hom Female vs. Male p=0.7125
s-TBI<br>♂ (n = 3, WT)<br>♂ (n = 7, Hemi)<br>♂ (n = 7, Hom)<br>♀ (n = 7, WT)<br>♀ (n = 8, Hemi)<br>♀ (n = 7, Hom)	Interaction<br>Sex<br>Genotype	F(2, 32) = 3.018; p=0.0630<br>F(1, 32) = 2.784; p=0.1050<br>F(2, 32) = 37.05; **p<0.0001**	WT Female vs. Male p=0.9979<br>Hemi Female vs. Male p=0.9979<br>Hom Female vs. Male p=0.0135
